# Structural basis for ligand binding modes of CTP synthase

**DOI:** 10.1073/pnas.2026621118

**Published:** 2021-07-23

**Authors:** Xian Zhou, Chen-Jun Guo, Chia-Chun Chang, Jiale Zhong, Huan-Huan Hu, Guang-Ming Lu, Ji-Long Liu

**Affiliations:** ^a^School of Life Science and Technology, ShanghaiTech University, Shanghai, 201210, China;; ^b^CAS Center for Excellence in Molecular Cell Science, Shanghai Institute of Biochemistry and Cell Biology, Chinese Academy of Sciences, Shanghai, 200031, China;; ^c^University of Chinese Academy of Sciences, Beijing, 100049, China;; ^d^Department of Physiology, Anatomy and Genetics, University of Oxford, Oxford, OX1 3PT, UK

**Keywords:** CTP synthase, allosteric regulation, cryo–electron microscopy, cytoophidium

## Abstract

Cytidine triphosphate synthase (CTPS), which comprises an ammonia ligase domain and a glutamine amidotransferase domain, catalyzes the final step of de novo CTP biosynthesis. The activity of CTPS is regulated by the binding of four nucleotides and glutamine. While glutamine serves as an ammonia donor for the ATP-dependent conversion of UTP to CTP, the fourth nucleotide GTP acts as an allosteric activator. Models have been proposed to explain the mechanisms of action at the active site of the ammonia ligase domain and the conformational changes derived by GTP binding. However, actual GTP/ATP/UTP binding modes and relevant conformational changes have not been revealed fully. Here, we report the discovery of binding modes of four nucleotides and a glutamine analog 6-diazo-5-oxo-L-norleucine in *Drosophila* CTPS by cryo–electron microscopy with near-atomic resolution. Interactions between GTP and surrounding residues indicate that GTP acts to coordinate reactions at both domains by directly blocking ammonia leakage and stabilizing the ammonia tunnel. Additionally, we observe the ATP-dependent UTP phosphorylation intermediate and determine interacting residues at the ammonia ligase. A noncanonical CTP binding at the ATP binding site suggests another layer of feedback inhibition. Our findings not only delineate the structure of CTPS in the presence of all substrates but also complete our understanding of the underlying mechanisms of the allosteric regulation and CTP synthesis.

## Significance

In the current study, we successfully push the resolution to near-atomic levels (2.48 Å) to analyze the polymer structure of *Drosophila melanogaster* cytidine triphosphate synthase (dmCTPS) with all its substrates (6-diazo-5-oxo-L-norleucine being used to represent glutamine). We have precisely located all ligands in a CTPS structure with a solid electron density map. With this model, we present a structural conformation of the GTP binding site and demonstrate its roles in mediating glutamine binding, NH_3_ transport, and stabilizing the ammonia tunnel. Additionally, the intermediate in the ATP-dependent phosphorylation reaction is observed allowing us to identify the residues participating in catalysis.

Cytidine triphosphate synthase (CTPS) catalyzes the final and rate-limiting step of de novo CTP biosynthesis, in which a UTP is converted into CTP with the consumption of an ATP and a glutamine. As its product CTP is required for DNA, RNA, and phospholipid synthesis, CTPS plays a critical role in fueling active cell metabolism especially in the cases of proliferative cells, such as lymphocytes and certain cancers, in which CTPS expression and activity are up-regulated (1–4). Therefore, CTPS has long been considered as a potential drug target for diseases including parasitic infections, viral infections, and cancers (5, 6).

A CTPS protein comprises two domains, the N-terminal ammonia ligase (AL) domain and the C-terminal glutamine amidotransferase (GAT) domain (7). While the active site at the AL domain activates UTP by phosphorylation using ATP to generate an iminophosphate intermediate that can react with ammonia to yield CTP, the GAT domain mediates the hydrolysis of glutamine to yield NH_3_ with GTP as allosteric activator (8, 9). The nascent ammonia is delivered through an “ammonia tunnel,” which connects the active sites at both domains, to complete the reaction (10). A few mechanisms are proposed from structural and biochemical studies to control the pace of the reaction, as follows. First, GTP appears to promote channeling of NH_3_ derived from glutamine hydrolysis to the synthase site by preventing the ammonia tunnel from being constricted or leaky (11–13). Second, the “gate” residue of the ammonia tunnel controls the access to the ALase active site, which is available to NH_3_ only when bound by UTP (10). And third, product CTP serves as a feedback inhibitor to CTPS by competitive binding at the UTP binding site (14, 15).

Despite around 70 y of study on CTPS, the details underlying ligand binding and conformational changes coordinating glutamine hydrolysis and CTP synthesis remain largely unclear. Previously, we used cryo–electron microscopy (cryo-EM) to resolve polymer structures of *Drosophila melanogaster* CTPS (dmCTPS) in its substrate-bound and product-bound states, providing basic structural information about a distinctive intracellular CTPS structure termed the cytoophidium in various organisms (16–23). Although we successfully revealed pivotal regions involving CTPS polymerization and assessed its effects on the catalytic activity, owing to the limit of resolution we were unable to explain molecular mechanisms underlying the reaction of CTPS.

In the current study, we resolved polymer structures of substrate- and product-bound dmCTPS at near-atomic resolution. With our models, binding modes of all ligands are precisely determined. Based on the conformational differences between substrate- and product-bound structures, we propose mechanisms of the coordination of glutamine hydrolysis and CTP synthesis at two separate domains by the allosteric regulator GTP. The phosphorylated UTP intermediate at the AL domain was observed, providing evidence for critical residues that participate in catalysis.

## Results

### Generation of Substrate- and Product-Bound *Drosophila* CTPS Tetramer Models at Near-Atomic Resolution.

**Fig. 1.**
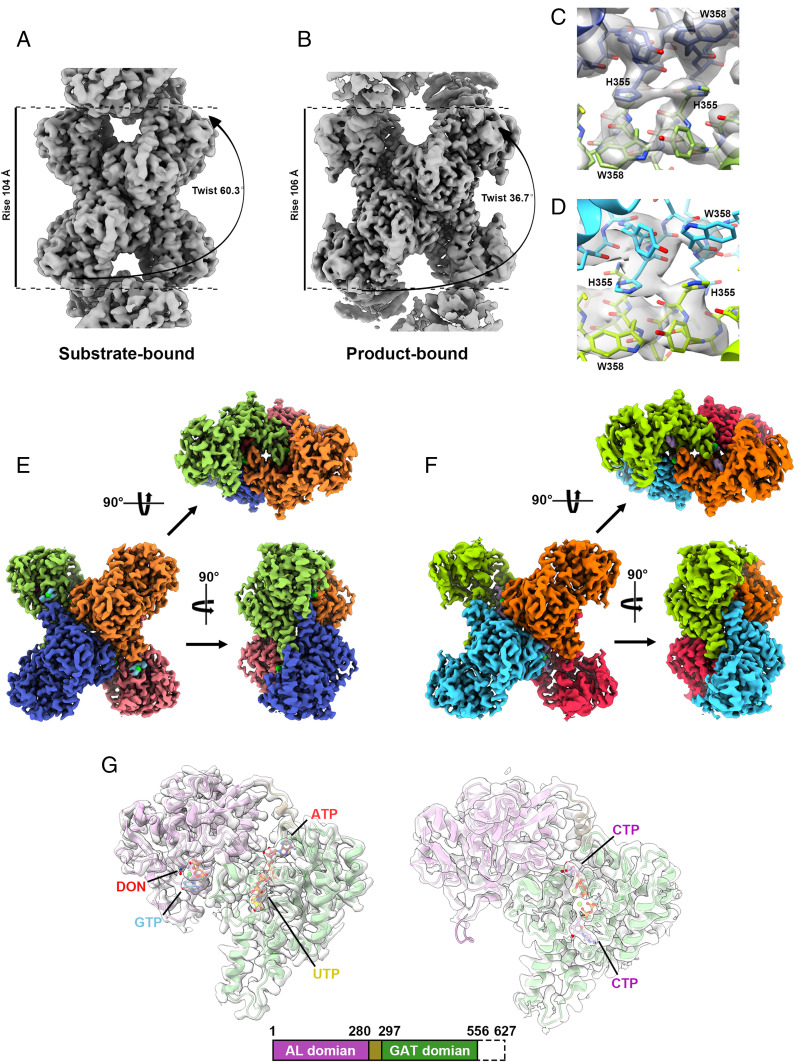
Overall structures of substrate-bound and product-bound *Drosophila* CTPS tetramer. (*A* and *B*) Cryo-EM reconstructions of substrate-bound (*A*, 2.48-Å resolution) and product-bound (*B*, 2.65-Å resolution) dmCTPS tetramers. (*C* and *D*) The models and maps of the tetramer–tetramer interaction interface of substrate-bound (*C*) and product-bound (*D*) dmCTPS polymers. (*E* and *F*) Models of substrate-bound (*E*) and product-bound (*F*) dmCTPS tetramers. (*G*) Models of dmCTPS monomers display binding site of each ligand in substrate-bound and product-bound states. Residues^556-627^ are disordered in the models.

The glutamine antagonist 6-diazo-5-oxo-L-norleucine (DON) inhibits glutamine metabolism and has shown robust anticancer efficacy in preclinical and clinical studies since the 1950s (24, 25). This diazo compound has a wide range of glutamine-utilizing proteins as targets, including CTPS. Due to the similarity to glutamine, it can enter glutamine binding sites of target proteins and inhibit their activities by covalent binding. In this study, we attempted to determine the molecular mechanisms of the catalytic action of CTPS by comparing conformations of dmCTPS in its substrate-bound and product-bound states at near-atomic resolution. To reveal the structure of dmCTPS bound by all ligands without completing the reaction (dmCTPS^+Sub^), we used DON as a glutamine substitute for cryo-EM. We selected 424,195 particles of full-length dmCTPS in the mixture of 10 mM Mg^2+^, 2 mM ATP, 2 mM UTP, 2 mM GTP, and 6 μM DON. For the product-bound state (dmCTPS^+Pro^), we imaged dmCTPS in the presence of 10 mM Mg^2+^ and 2 mM CTP and picked 1,563,553 particles. Consequently, we reconstructed dmCTPS^+Sub^ and dmCTPS^+Pro^ tetramer and polymer structures at 2.48 and 2.65 Å resolution (Fig. 1 and *SI Appendix*, Figs. S1–S6 and Table S1).

We have previously demonstrated that dmCTPS tetramers can assemble into polymers under the conditions with either substrate or product binding. With conditions described in the last paragraph, we first reconstructed dmCTPS polymer and tetramer in both states (Fig. 1 *A*, *B*, *E* and *F*). In these models, the interaction at the interfaces of two neighbor tetramers is clearly seen between His355 and Trp358 of the partner tetramers (Fig. 1 *C* and *D*), and the overall polymer structures have slight differences from our previous dmCTPS^+Sub^ and dmCTPS^+Pro^ models, as the twist and rise of the polymers are 60.3° and 36.7° and 104 and 106 Å, respectively (Fig. 1 *A* and *B*). The fashion of interaction between dmCTPS tetramers is very similar to hCTPS1 and hCTPS2 tetramers, which can also polymerize depending on the interaction between His355 and Trp358 (26, 27).

**Fig. 2.**
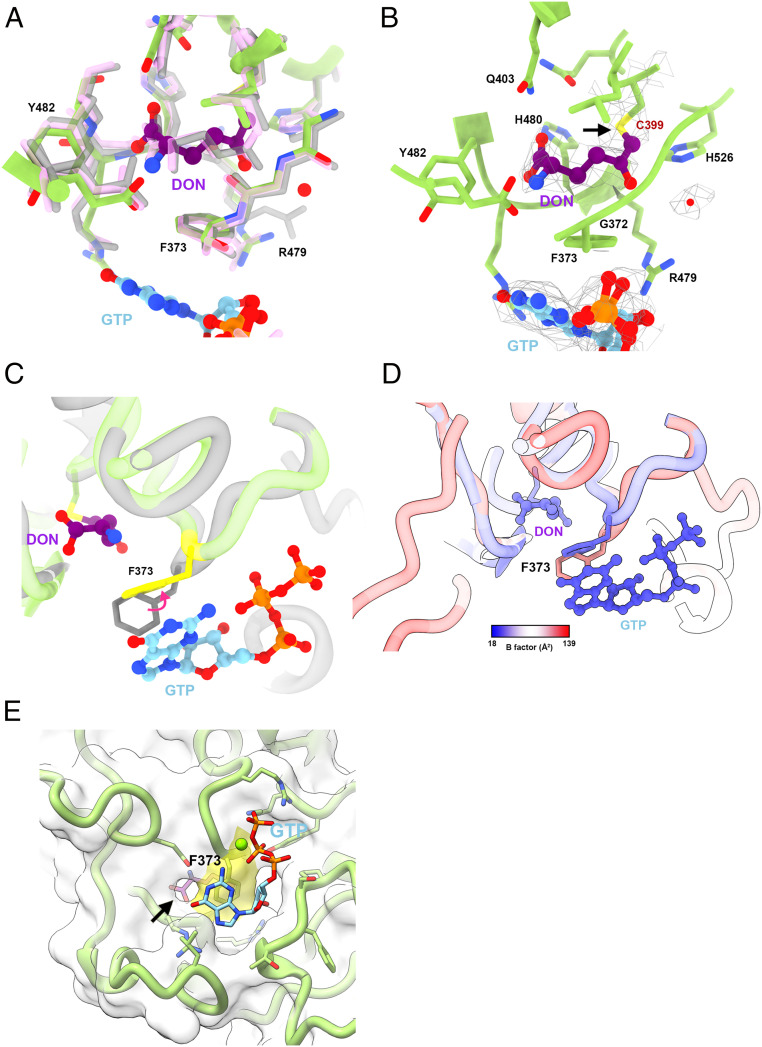
Conformational changes of Phe373 reveals its role in regulating the binding of glutamine and GTP. (*A*) The structural comparison of the glutamine binding sites in glutamine-bound *E. coli*. CTPS (5TKV, pink), *Thermus thermophilus* HB8 CTPS (1VCO, gray), and dmCTPS^+Sub^ (green) model. (*B*) DON is located at the glutamine binding site at the GAT domain. The electron density map demonstrates the covalent bond between DON and Cys399 (arrow). (*C*) The structural comparison of the Phe373 in dmCTPS^+Sub^ (green) and dmCTPS^+Pro^ (gray) models. The Phe373 is ordered and more conductive to stacking with guanine in the dmCTPS^+Sub^ state. (*D*) B factors are shown on the putative glutamine entrance in dmCTPS^+Sub^ and dmCTPS^+Pro^ models. (*E*) The Phe373 (yellow) is located at the putative entrance (arrow) to the glutamine binding site, which is adjacent to the GTP binding site. In *A*, *C*, and *D*, we aligned the GAT domain (297 to 556) for comparison.

DON and GTP, which were not present in the previous model, are located at the GAT domain of the dmCTPS^+Sub^ (Fig. 1*G*). The glutamine binding mode of CTPS has been revealed with a few prokaryotic CTPS models (27, 28). In our dmCTPS^+Sub^ model, the binding mode of DON shows high consistency with glutamine binding in prokaryotic CTPS models (Fig. 2*A*), and the covalent bond between Cys399 and DON is observed (Fig. 2*B*), indicating that the glutamine binding site is active for the reaction of the thiol nucleophile. Hence, our model fully displays the dmCTPS structure with all substrates bound. We compared the differences in the position of residues involved in DON/glutamine and GTP binding in dmCTPS^+Sub^ and dmCTPS^+Pro^ models and found Phe373 moves greatly upon DON and GTP binding (Fig. 2*C*). Notably, the density for Phe373 in maps of dmCTPS^+Pro^ and hCTPS2 (6PK4, bound with ATP and UTP) shows the high flexibility of the residue. To elucidate if Phe373 could be stabilized by DON/glutamine alone, we analyzed the maps of published *Thermus thermophilus* CTPS (ttCTPS) structures in its apo- (1VCM) and glutamine-bound (1VCO) conformations (28). While the density of Phe373 (Phe365 in ttCTPS) is not complete in the apo state, the model of Phe373 could be well fitted into the map in the glutamine-bound conformation (*SI Appendix*, Fig. S7*A*). These correspondences suggest that Phe373 conformational differences between dmCTPS^+Sub^ and dmCTPS^+Pro^ models are due to interactions with bound DON/glutamine (Fig. 2*D* and *SI Appendix*, Fig. S7*A*). Considering Phe373 is located at the entrance of the glutamine binding site (Fig. 2*E*), we propose that its flexibility in unbound structures may facilitate glutamine entry, and its ordering in the bound structures indicates a stabilizing interaction.

### Near-Atomic Model of Substrate-Bound dmCTPS Reveals the GTP Binding Mode.

**Fig. 3.**
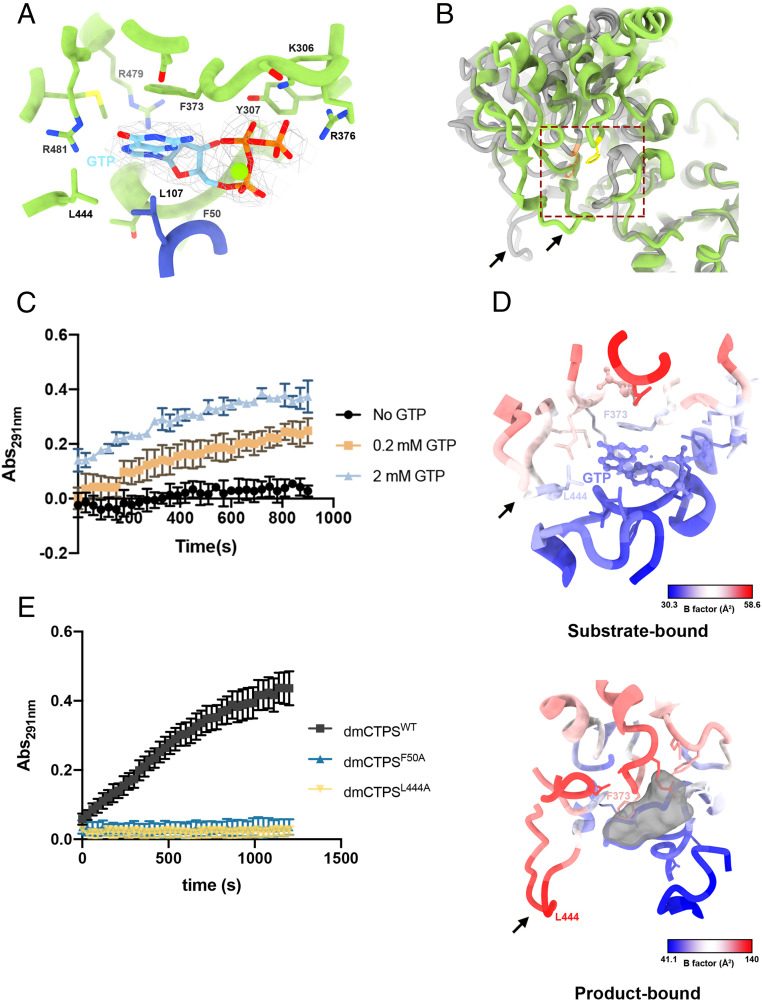
Determination of GTP binding site and relevant conformational changes reveal the mechanism of allosteric regulation. (*A*) The GTP binding site at GAT domain. Residues interacting with GTP are indicated. (*B*) The structure comparison of the GTP binding sites in dmCTPS^+Sub^ (green) and dmCTPS^+Pro^ (gray) models shows the general conformational change of the GAT domain and the switch of the wing region. Phe373 is shown in yellow and orange in dmCTPS^+Sub^ and dmCTPS^+Pro^ models, respectively. The wing structure is indicated by arrows. The AL domain (1 to 280) was used for the alignment. (*C*) Analysis for generation of CTP by wild-type dmCTPS (dmCTPS^WT^) in conditions with 0, 0.2, and 2 mM GTP. The absorption of 291 nm (Abs_291nm_) represents the concentration of CTP in samples. For the analysis, 2 μM dmCTPS was mixed with 2 mM UTP, 2mM ATP, 10 mM MgCl_2_, 25 mM Tris HCl (pH 7.5), and GTP at different concentrations before the supplement of 10 mM glutamine for initiating the reaction. (Error bars, SD.) (*D*) B factors are shown on GTP binding sites in dmCTPS^+Sub^ and dmCTPS^+Pro^ models. Arrows indicate the wing structure. (*E*) Analysis for generation of CTP by mutant dmCTPS (F50A and L444A) in condition with 0.2 mM GTP.

Although GTP has been shown to have a great influence on the catalytic function of CTPS, its binding site and associated conformational changes are not yet determined with actual models. By using DON as a glutamine substitute in the mixture of substrates, we successfully reconstructed a CTPS map with actual GTP binding. Our result precisely positions the GTP binding site at dmCTPS and demonstrates residues responsible for GTP affinity (Fig. 3*A*). The GTP binding site is located at a cleft between the GAT and AL domain, which is similar to the predicted GTP binding position in ecCTPS (10). It was shown in previous studies that the CTPS structure displays a relatively open state when it is bound with CTP (14, 26, 27). Interestingly, another model of *Mycobacterium tuberculosis* CTPS with two UTP molecules, one at the UTP binding site while the other sits at the ATP binding site, is also in its open state (29). Therefore, we propose that this open-to-closed structural transition is likely due to binding of ligands at the AL domain, as previously suggested (26, 27). The distance between both sides of the cleft varies greatly in closed and open states of CTPS, which are modulated by the binding of ligands at the AL domain (Fig. 3*B*). These suggest that binding of ATP and/or UTP triggers the open-to-closed transition and thereby alters the conformation of the cleft to create the GTP binding site. Conversely, while CTPS returns to the open state, the GTP can no longer bind with CTPS.

At the GTP binding site, the guanine base of GTP interacts with Leu444 of the binding monomer and Leu107 of another monomer through dispersion interactions and forms three hydrogen bonds with the side chain of Arg481. The oxygen of the guanine base interacts with the ε-nitrogen of Arg481 through a hydrogen bond, which might be the key to distinguish between GTP and ATP at the binding site (Fig. 3*A*). In addition, the π–π interaction between the guanine ring and Phe373 also contributes to the binding (Fig. 3*A*). According to the comparison of ttCTPS structures in its apo and glutamine-bound states and our models, the binding of DON/glutamine stabilizes Phe373, making it more conducive to stacking with guanine (Fig. 2*C*). The π–π interaction between Phe373 and GTP, which may stabilize GTP binding, might be the key to holding the GTP at the binding site under observation. That is, the presence of glutamine or glutamine analogs at the binding site is a prerequisite for the observation of bound GTP. The lack of this interaction might be the reason why the bound GTP had not previously been observed in the conditions with GTP but not glutamine or glutamine analogs.

The ribose ring of GTP interacts with Arg479 and Phe50 through hydrogen bonds between the O2 and O3 and the amino acid, respectively. The triphosphate part also forms three hydrogen bonds with Lys306, Tyr307, and Arg376, generating strong bonds between GTP and the protein (Fig. 3*A*). GTP is known as an allosteric activator of the GATase activity of CTPS. Our data also indicate that GTP is required for dmCTPS to catalyze Gln-dependent CTP synthesis (Fig. 3*C*). Previous biochemical research has demonstrated that point mutations at Arg359 of *Lactococcus lactis* CTPS (llCTPS) and Leu109 of *Escherichia coli* CTPS (ecCTPS) greatly impede the Gln-dependent CTP synthesis of CTPS in the presence of GTP (12, 30). The correspondent residues of these two points of dmCTPS are Arg376 and Leu107, both of which are shown to directly interact with GTP in our model (Fig. 3*A*). This consistency with previous reports reinforces our findings about the GTP binding mode.

### The Flexible “Wing” Structure Plays Pivotal Role in Regulating GTP Binding and CTP Synthesis.

Furthermore, GTP-interacting residues in our model are conserved across different CTPS isoforms of species from *E. coli* to eukaryotes. The exception is Leu444, at which position of human CTPS1 (hCTPS1) and llCTPS is a methionine. Interestingly, the Leu444 is located at the middle of a loop^440-448^ of the GAT domain, referred to as the “wing” from here on (Fig. 3*B*). This wing structure was predicted to be very flexible, and so it was not clearly resolved in previous CTPS models. The wing is also flexible in our dmCTPS^+Pro^ model (Fig. 3*D*). Yet, stabilized by the interaction between Leu444 and GTP, the wing is rather rigid in our dmCTPS^+Sub^ map (Fig. 3*D*). This interaction brings the wing swinging toward an opening of the pocket of the glutamine binding site. However, it is uncertain if this conformational change would affect glutamine binding.

Apart from GTP, Leu107 is also predicted to interact with Leu444 by a dispersion interaction. Since Leu107 mutation had been shown to greatly impair GTP binding in a previous study, we wondered if Leu444 also plays a pivotal role in stabilizing GTP binding. To this end, we generated L444A mutant dmCTPS for the activity analysis. We predicted that the loss of Leu444 would make Leu107 and the binding of GTP unstable and so attenuate the activity of CTPS. As a result, the mutant dmCTPS displayed an extremely low activity, which is similar to Leu109 mutation in ecCTPS or the reaction conditions with no GTP (Fig. 3*E*). We also used higher GTP concentration (2 mM) in the analysis for CTP production and observed only slightly increased activity of both mutants (*SI Appendix*, Fig. S8). These results suggest that the binding of GTP is relatively weak but critical to the reaction as the loss of single interaction could lead a dramatic decrease in the activity of the enzyme. Although, this could also be a consequence of failure of required conformational changes for the catalytic reaction.

### GTP Binding Mode Suggests Its Role in Preventing Leakage of Nascent Ammonia.

**Fig. 4.**
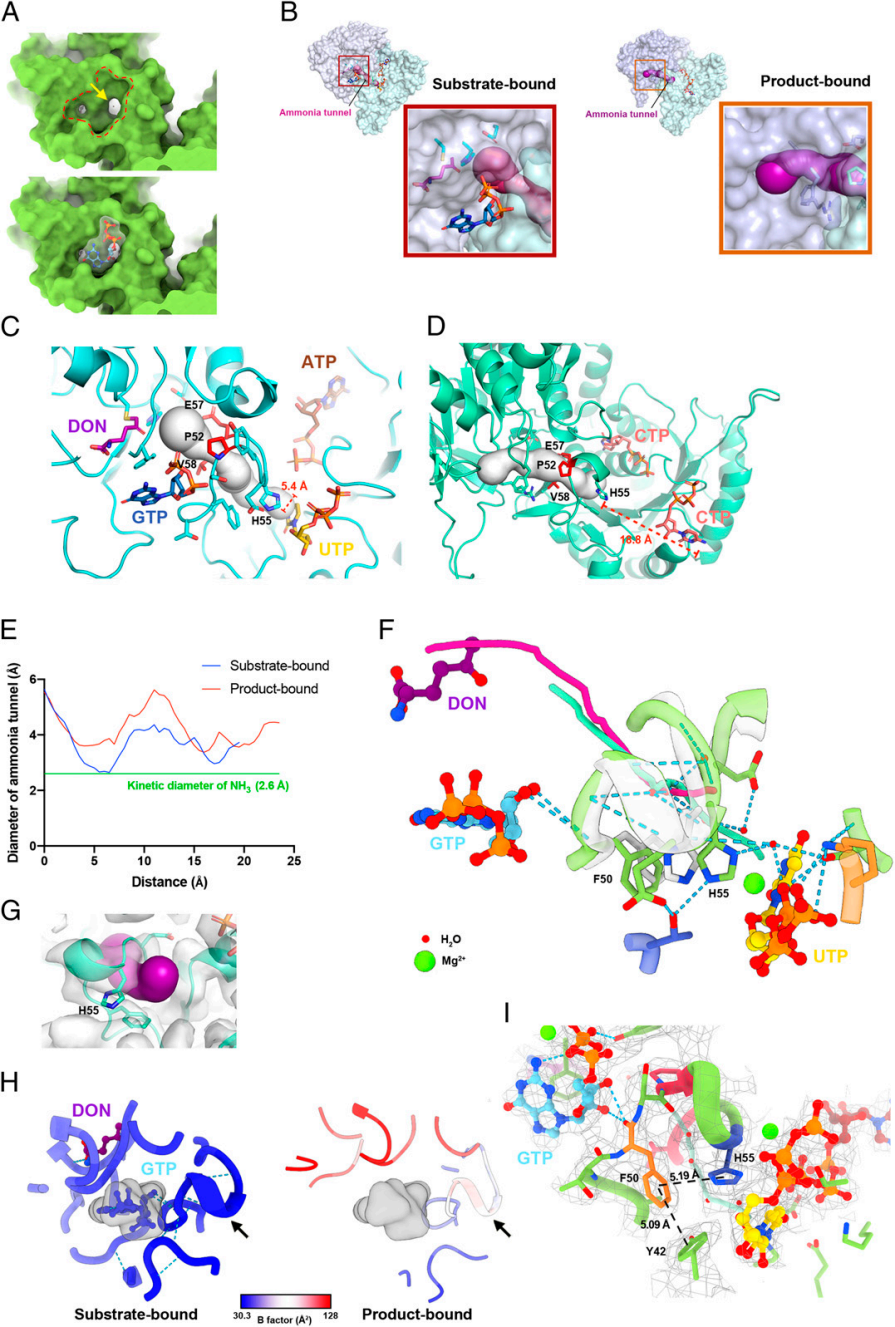
Conformational changes of ammonia tunnel reveals its open states in both substrate-bound and product-bound dmCTPS. (*A*) Protein surface of the GTP binding cleft (selected by red dashed line) between the GAT domain and AL domain. An opening (yellow arrow) connecting the active site of the GAT domain and the ammonia tunnel would be fully covered by GTP. (*B*) Overview of the ammonia tunnel in substrate- and product-bound dmCTPS. The estimated tunnel is shown in magenta. (*C* and *D*) Models showing the ammonia tunnel and surrounding residues in (*C*) substrate- and (*D*) product-bound states. The tunnel is displayed by serial white spheres. The size of each sphere represents the estimated space in the tunnel at each point. The distance (dashed lines) is shown between the His55 side chain and uracil O4 and cytosine NH_2_. (*E*) Graph of the diameter of the ammonia tunnel along its length in substrate- and product-bound dmCTPS. (*F*) The structural comparison of the ammonia tunnel structures in dmCTPS^+Sub^ and dmCTPS^+Pro^ models. The estimated routes of the tunnel in dmCTPS^+Sub^ and dmCTPS^+Pro^ models are shown in cyan and magenta, respectively. The region of ammonia tunnel (50 to 58) was used for superposition. (*G*) Electron density map shows that the putative “gate” His55 does not enclose the ammonia tunnel in the dmCTPS^+Pro^ state. Estimated interior space of ammonia tunnel is displayed in magenta. (*H*) B factors are shown on ammonia tunnels (arrows) in dmCTPS^+Sub^ and dmCTPS^+Pro^ models. (*I*) The interactions of Phe50 with Tyr42 and His55 may contribute to the stabilization of the ammonia tunnel. The distance between aromatic rings is shown.

There are two openings for the glutamine binding site of the GAT domain (Fig. 4*A*). The second one faces one end of the ammonia tunnel, and this is believed to be the route for nascent NH_3_ proceeding to the next reaction (10). According to all published CTPS models of various species, a cleft, where now we confirm the GTP binding site is located, between the glutamine hydrolysis active site and the entrance of the ammonia tunnel is not enclosed by the protein structure (10, 26–28) (Fig. 4 *A* and *B*). Consequently, nascent NH_3_ from the active site may escape through the cleft.

Conversely, as suggested by a previous study, exogenous NH_3_ may also enter the ammonia tunnel through this locus and be utilized for NH_3_-dependent CTP synthesis (10). However, when GTP is positioned at the binding site, the cleft between the glutaminase active site and the ammonia tunnel is fully covered by GTP, suggesting that GTP serves as a block to prevent molecules shuttling between the interior and exterior spaces of the protein (Fig. 4 *A* and *B*). Indeed, point mutations that impair GTP binding have been shown to lead to a decrease in activity and uncoupling between the NH_3_ derived from glutamine hydrolysis and CTP formations (12, 30, 31). Moreover, it has been demonstrated that GTP inhibits NH_3_-dependent CTP synthesis in a concentration-dependent manner in the absence of glutamine, supporting the hypothesis that exogenous NH_3_ may enter the tunnel through the GTP binding site (13, 32).

### Conformational Switch of Ammonia Tunnel Offers Insight into Coordination between AL Domain and GAT Domain.

Class I glutamine-dependent amidotransferases, including CTPS, have tunnels to facilitate the transfer of nascent NH_3_ (33). In our dmCTPS^+Sub^ tetramer model, the length of the ammonia tunnel is around 19.5 Å, and its diameter ranges from 2.6 to 4.4 Å. In the dmCTPS^+Pro^ tetramer, the length is around 23.5 Å, and the diameter ranges from 3.38 to 5.54 Å (Fig. 4 *C*–*E*). In both states, the size of the tunnel is sufficient for NH_3_ transport, as the kinetic diameter of NH_3_ is estimated to be 2.6 Å, a similar size to water. Pro52, His55, and Val58 in the tunnels of hCTPS2 and ecCTPS are proposed to block NH_3_ transport (10, 26).

We also observed a bottleneck of the tunnel at the locus surrounded by Pro52, Glu57, and Val58 (Fig. 4*E*). However, our measurement suggests that this is not narrow enough to completely stop NH_3_ passing through. In addition, six water molecules are present in the ammonia tunnel in both dmCTPS^+Sub^ and dmCTPS^+Pro^ structures, indicating that the tunnel is permeable to both ammonia and water (Fig. 4*F*).

On the other hand, His55 (His57 of ecCTPS) was predicted to act as a gate at the exit of the ammonia tunnel (10, 13, 17, 26). It was suggested that when CTPS is bound with substrates, the interaction between UTP and His55 alters the orientation of His55, causing the gate to open. Our map confirms the interaction between His55 and the α-phosphate of UTP, which is stabilized by a Ser12-interacting water molecule (Fig. 4*F*). Yet, the exit of ammonia is not closed in the dmCTPS^+Pro^ map (Fig. 4*G*). We analyzed the conformational change at the UTP binding site and determined that the distance from CTP to the exit of the tunnel is about 18.8 Å in the dmCTPS^+Pro^, whereas UTP in dmCTPS^+Sub^ sits right before the opening at a distance of about 5.4 Å (Fig. 4 *C* and *D*). In two reported *Mycobacterium tuberculosis* CTPS models, one is bound with two UTP molecules (4ZDJ) and the other is bound with UTP, ACP, and DON (4ZDK); the UTP is present at the binding site of the CTPS in open state. We compared their models with our dmCTPS^+sub^ and determined that the interaction between His55 and UTP only occurs when the CTPS is in its closed state (*SI Appendix*, Fig. S9). These suggest that the open-to-closed transition brings the UTP toward the exit of ammonia tunnel, making it more accessible to the coming NH_3_.

We noticed that the interior part of the dmCTPS ammonia tunnel is less stable without the presence of DON/glutamine and GTP according to B factors of the models (Fig. 4*H* and *SI Appendix*, Fig. S7*B*). When DON/glutamine and GTP are unbound, three GTP-interacting regions, residues^50-58^, residues^306-310^, and residues^371-377^, become more flexible (Fig. 4*H*). Of them, residues^50-58^ directly form a part of the tunnel (Fig. 4 *C* and *D*). GTP interacts with the backbone of Phe50 (Fig. 4*I*). We speculated that the tunnel structure could be stabilized by GTP through this interaction and also π–π stacking interactions of Phe50 with His55 and Tyr42 (Fig. 4*I*). We generated F50A mutant dmCTPS for enzyme activity analysis to access the importance of the role of Phe50. Similar to other point mutations disrupting the interaction between CTPS and GTP, F50A shows nearly no activity (Fig. 3*E*). Although it is uncertain the impaired activity is caused by the loss of GTP binding affinity or the conformational change of ammonia tunnel, our data suggests that these interactions with Phe50 are critical for CTP synthesis.

### Electron Density Map of dmCTPS Gives Evidence of Residues Catalyzing UTP Phosphorylation.

**Fig. 5.**
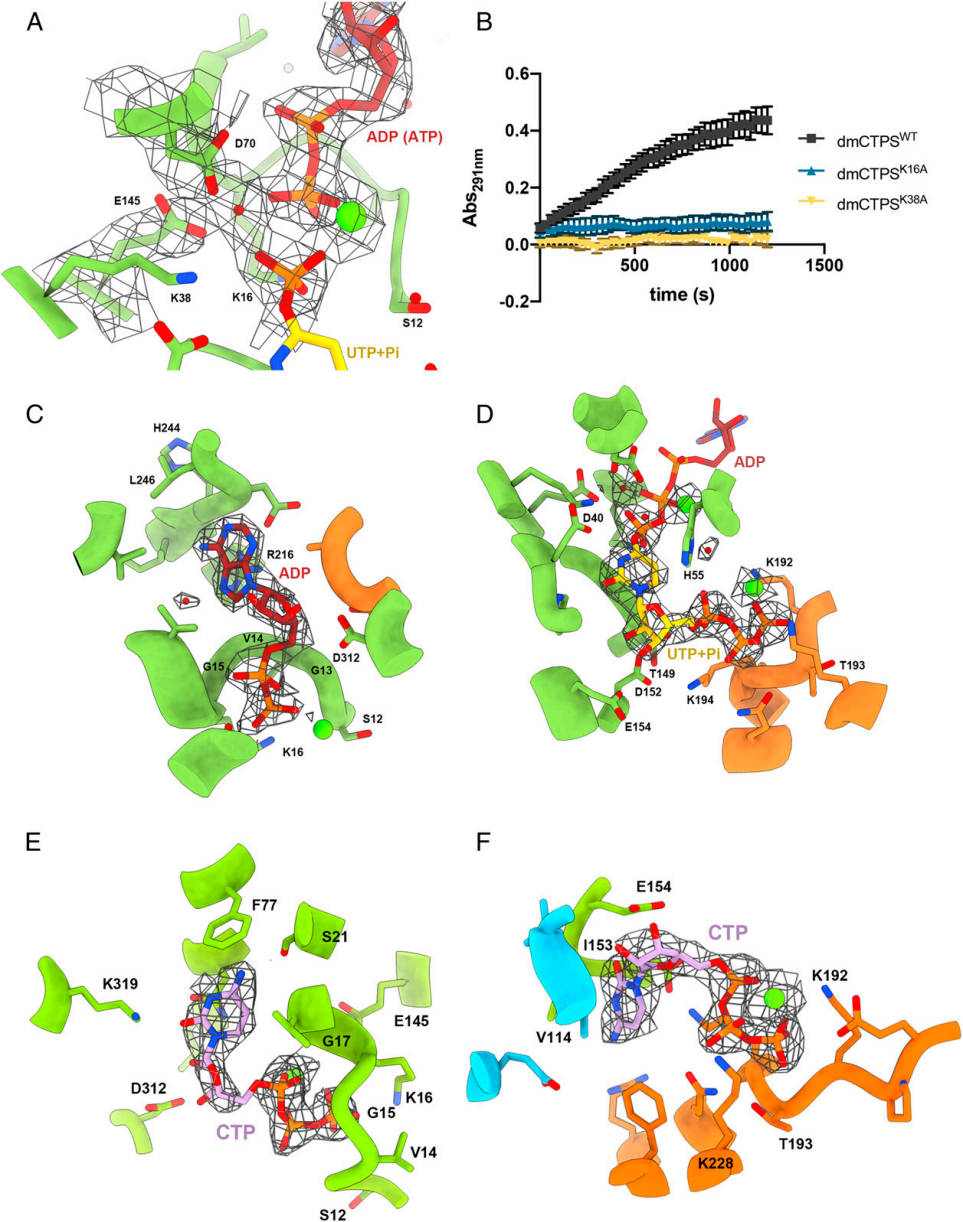
Ligands binding at AL domain reveal mechanisms of UTP phosphorylation and feedback inhibition. (*A*) Electron density map showing the mechanism of UTP phosphorylation. (*B*) Analysis for generation of CTP by K16A and K38A mutant dmCTPS in condition with 0.2 mM GTP. The absorption of 291 nm (Abs_291nm_) represents the concentration of CTP in samples. For the analysis, 2 μM dmCTPS was mixed with 2 mM UTP, 2 mM ATP, 10 mM MgCl_2_, 25 mM Tris HCl (pH 7.5), and 0.2 mM GTP. Glutamine (10 mM) is supplied for initiating the reaction. (Error bars, SD.) (*C* and *D*) The ATP (*C*) and UTP (*D*) binding site at AL domain. ATP and UTP have become ADP and phosphorylated UTP in our dmCTPS^+Sub^ model. Residues interacting with ADP/UTP are indicated. (*E* and *F*) The competitive binding of CTP with ATP (*E*) and UTP (*F*). Water molecules are shown as red spheres in all panels.

The residues responsible for catalyzing the first reaction at the AL domain have been predicted by a computational structural comparison between ecCTPS and other amidoligases but have not been experimentally confirmed yet (10). In our dmCTPS^+Sub^ map, we observe additional electron density merging with the uracil base in the direction of the 4-oxygen atom, which would be phosphorylated in the reaction. Meanwhile, the signal of the γ-phosphate of ATP is missing, suggesting that the reaction of UTP phosphorylation is taking place in our imaging condition. (Fig. 5*A* and *SI Appendix*, Fig. S10). Furthermore, an electron density signal, which is proposed to be a magnesium, is located between the β-phosphate of ATP and the phosphate on the uracil (Fig. 5*A*). This magnesium is possibly responsible for activating transfer of the γ-phosphate of ATP as predicted previously (10).

We further focused on residues participating in this transformation and identified Lys16 and Lys38 directly interact with transferred phosphate and Asp70 and Glu145 also interact through a water molecule, supporting previous predictions about their function of stabilizing the anionic transition state during the reaction (10) (Fig. 5*A*). We sought to examine whether our model for catalysis is accurate by monitoring CTP production by K16A and K38A mutant dmCTPS. As a result, both mutants displayed dramatically reduced enzyme activity, indicating that both Lys16 and Lys38 are involved in the reaction (Fig. 5*B*).

We reveal that the binding of ATP, which became ADP in our model, relies on hydrogen bonds between the adenine base and His244 and Leu246 interactions between ribose and Gly13, Val14, Arg216, and Asp312 and the interactions between phosphate and three residues, Gly13, Gly15, and Lys16, on an α-helix (Fig. 5*C*). Binding of UTP, which is merged with a phosphate at 4-oxygen in our model, is directed by hydrogen bonds between Asp40 and the uracil base and between Asp152, Glu154, and ribose. The triphosphate of UTP directly interacts with Lys192, Thr193, and Lys194 and also indirectly interacts with some other residues including Ser12, His55, Gln112, Thr149, Asp152, and Lys194 through water molecules (Fig. 5*D*). By showing the high structural similarity at the AL domain active sites of our dmCTPS^+Sub^ model and a substrate-bound hCTPS2 model, our data indicates that the ATP/ADP and UTP binding modes are very conserved between *Drosophila* and human CTPS (26) (*SI Appendix*, Fig. S11*A*).

### Noncanonical CTP Binding Site Overlaps with ATP Binding Site on AL Domain.

The product CTP can negatively regulate CTPS activity by competing for the binding site with UTP, leading to a balance of nucleotide levels. A point mutation, E161K of hCTPS (E160K in dmCTPS), which impairs CTP binding, can dramatically promote CTP synthesis even in the presence of a high level of CTP (34, 35). This feedback inhibition is suggested to be reduced by CTPS polymerization in human and *Drosophila* models (17, 27).

Consistent with previous models, CTP overlapped with UTP at binding sites with slight deviation (14, 26, 36) (Fig. 5*E*). To our surprise, we identified a second CTP molecule in an individual subunit of the dmCTPS^+Pro^ tetramer, whose binding site overlaps with ATP/ADP (Fig. 5*F*). In a product-bound hCTPS2 (6PK7) model, the same position was depicted to be an ADP (26) (*SI Appendix*, Fig. S11 *B* and *C*). Since CTP is the only nucleotide present under the conditions of analysis, it is unlikely that another molecule could be mistaken for CTP in our map. In addition, the electron density agrees very well with the CTP structure, confirming our findings of the CTP binding site of CTPS and also raising the possibility that binding competition, which inhibits the activity of CTPS, may occur at both the UTP and ATP binding sites. In fact, CTP is known to compete with ATP for the same binding site on aspartate transcarbamoylase (ATCase) (37), supporting the possibility of similar competition on CTPS. However, whether this competition between CTP and ATP on CTPS could occur under physiological CTP concentrations is yet to be determined.

## Discussion

To summarize the history of the study of CTPS, we have learned that this enzyme is highly conserved across species in many aspects including the reaction mechanisms and regulations, despite limited sequence variation (*SI Appendix*, Fig. S12). The full-length CTPS structure was first resolved with X-ray crystallography in the early 2000s, in two independent studies of ecCTPS and *Thermus thermophilus* CTPS tetramer structures in their sulfate-bound apo state (10, 28). The glutamine-bound and CTP-bound states were also depicted at a similar time (14, 28). The binding sites of all its ligands were predicted based on these models, and some critical residues have further been confirmed biochemically. These findings have become the basis of CTPS-related studies in all fields. Nonetheless, some important information is not available from the study of the crystal structure due to the limitations of the technique; for example, failure to crystalize target protein with desired ligand combinations.

Recent progress in cryo-EM and computation have improved the resolution to approaching the atomic level. Because of this advance, we are now able to examine protein structures under more diverse conditions. In the current study, we successfully pushed the resolution to 2.48 Å, at near-atomic level, to analyze the polymer structure of dmCTPS with all its substrates (DON being used to represent glutamine). In this study, it has been possible to precisely locate all ligands in a CTPS structure with a solid electron density map.

With this model, we successfully revealed the exact GTP binding site on the GAT domain. Formation of this GTP binding site could be initiated by the binding of UTP and ATP, and the glutamine at the binding site further stabilizes GTP binding through moderating the rotation of Phe373. A flexible “wing” region on the GAT domain becomes stable upon GTP binding in our model. Leu444 at the wing region is shown to contribute to the binding of GTP, and the interaction between Leu444 and GTP may stabilize the wing structure. A L444A point mutation that disrupts this interaction results in a dramatic loss of catalytic activity, suggesting the GTP binding is relatively weak but critical to the reaction (Fig. 3*E*). Although, we could not completely rule out that such mutation may obviate appropriate conformational changes from occurring, thereby abrogating the catalytic reaction.

We also demonstrated the roles of GTP in mediating NH_3_ transport and stabilizing the ammonia tunnel (Fig. 4 *A*, *B,* and *H*). Second, as described earlier, how the ammonia tunnel is regulated during CTP synthesis is still controversial. Some studies suggest that His55 acts as the gate between the tunnel and the reaction site (10, 13, 17, 26). Our models also display the interaction between UTP and His55. However, the exit of the tunnel remains open without the interaction, indicating that the alteration of His55 would not block the tunnel (Fig. 4 *C* and *F*).

Endrizzi et al. have predicted the residues involved in catalysis at the ALase active site by comparison of the structure of ecCTPS with other enzymes catalyzing a similar chemical transformation (10). They suggested that Lys18, Lys40, Asp72, Glu140, and Gly143 of ecCTPS and a magnesium would surround the ATP and catalyze the reaction. Surprisingly, the electron density map of dmCTPS^+Sub^ showed 4-phospho-UTP intermediate under our imaging conditions, allowing us to capture the full reaction in situ. We found that residues Lys16, Lys38, Asp70, and Glu145 of dmCTPS did interact with ATP directly or through a water molecule, confirming their roles in the catalytic process (Fig. 5*A*). In contrast, the dmCTPS^+Pro^ model represents an inhibited state of dmCTPS. In this conformation, the UTP binding site is occupied by CTP as a well-known competitive inhibitor (Fig. 5*E*). However, the unexpected finding of a CTP molecule siting at the ATP/ADP binding site of the map gives rise to the notion that CTP may block CTPS by competing with UTP and also with ATP, suggesting an added level of complexity of feedback inhibition (Fig. 5*F*).

**Fig. 6.**
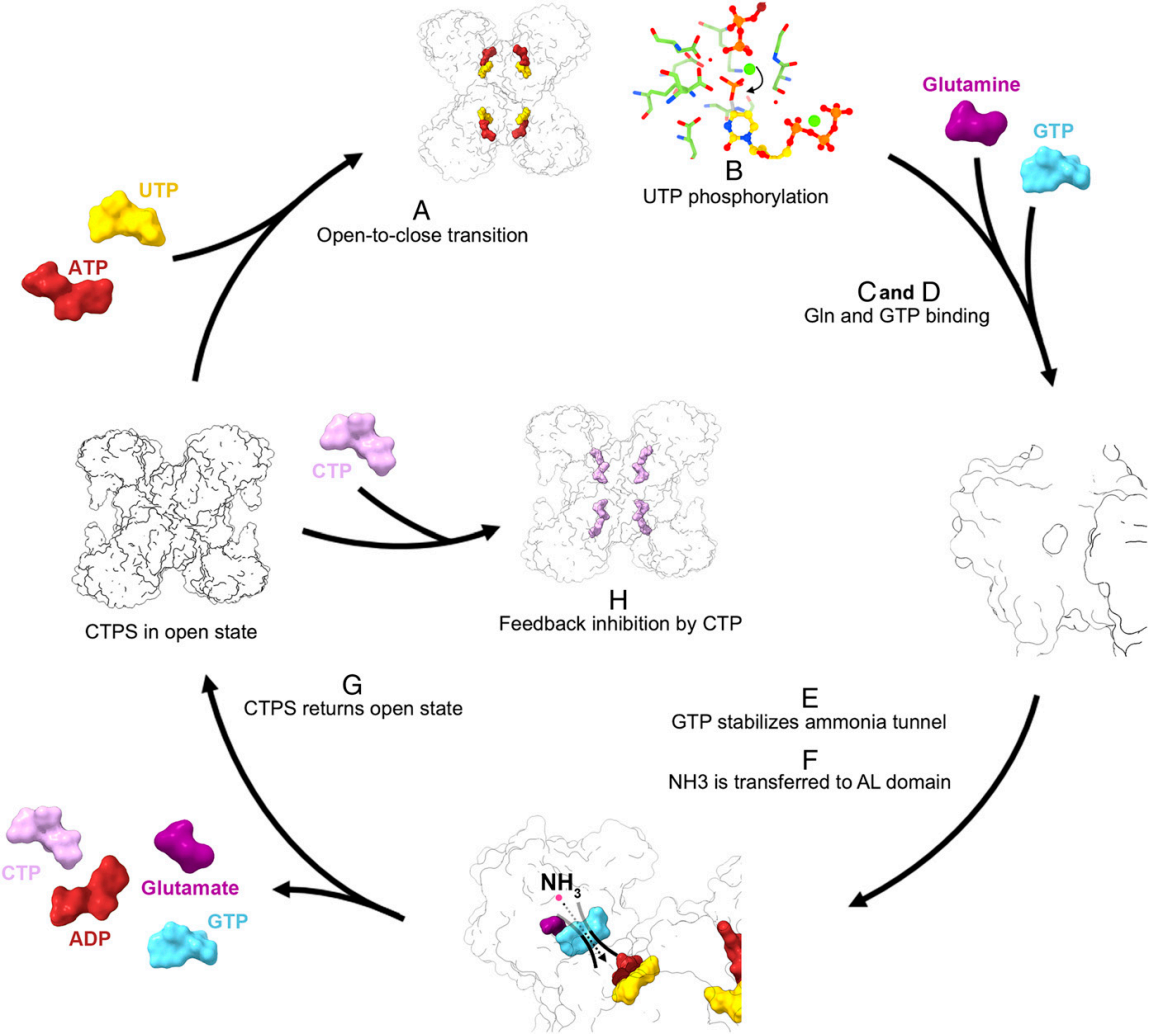
Model of conformational changes of dmCTPS mediating glutamine-dependent CTP synthesis. (*A*) The binding of ATP/UTP results in an open-to-close state transition. The cleft between GAT domain and AL domain contracts to form the GTP binding site. (*B*) The γ-phosphate of ATP is transferred to the 4-oxygen atom of the uracil base. (*C*) Glutamine enters the binding site and stabilizes Phe373. (*D*) GTP enters the binding site, and the binding is further stabilized by Phe373 and also Leu444 on the wing structure. (*E*) GTP covers the gap between the active site of the GAT domain and stabilizes the ammonia tunnel. (*F*) Nascent NH_3_ is transported to the AL domain through the tunnel and interacts with the iminophosphate intermediate to form CTP. (*G*) Ligands detach from binding sites, and CTPS returns to the open state. (*H*) CTP binds CTPS to competitively inhibit the binding of ATP and UTP.

Based on the structural comparison and the GTP binding mode, we propose that the full picture of mechanisms of glutamine-dependent CTP synthesis starts with the open-to-closed state transition, which is mediated by the binding of ATP and/or UTP (Fig. 6). This conformational change creates the GTP binding site at the GAT domain, allowing the binding of GTP. The binding of GTP could be further stabilized by the glutamine binding–mediated interaction with Phe373. Although the binding of glutamine and glutamine hydrolysis may occur even without GTP binding, the binding of GTP is required for the complete and stable ammonia tunnel. Consequently, the nascent NH_3_ is able to migrate to the active site of the AL domain without leakage. When the ATP and UTP are converted into ADP and CTP, the CTPS returns to its open state, ready for the next round of reactions (Fig. 6 and Movie S1).

Taken together, our dmCTPS reconstructions reach the near-atomic level, allowing us to actually visualize conformational changes between these two dmCTPS states. By comparing two structures, we demonstrate the relevant conformational changes at the GTP binding site, ammonia tunnel, and UTP active site. Based on our structural evidence, we propose a model to describe the regulation and coordination of reactions that occur at two distinct domains of CTPS. Nevertheless, more potential applications of our full-length CTPS models are still to be explored.

## Methods

### dmCTPS Expression and Purification.

A full-length *Drosophila* CTPS sequence was constructed with a C-terminal 6XHis-tag and driven by T7 promoter and transformed into *Transetta* (DE3) cells for expression. This was induced with 0.1 mM IPTG at 16 °C, overnight incubation. Cells were pelleted by centrifugation at 4,000 rpm for 20 min followed by resuspension in cold lysis buffer containing 150 mM NaCl, 50 mM Tris HCl (pH7.5), and 10% glycerol. The cell lysate was then centrifuged (18,000 rpm) at 4 °C for 40 min. Supernatant was collected and incubated with equilibrated Ni-Agarose (Qiagen) for 1 h. Subsequently, Ni-Agarose was washed with lysis buffer (20 mM Tris⋅HCl [pH 7.5], 150 mM NaCl, 10 mM imidazole, 1 mM PMSF, 1 mM leupeptin, 1 mM pepstatin, 1 mM benzamidine, and 1mM β-Me, and protein was then eluted with lysis buffer supplemented with 250 mM imidazole and 1 mM β-Me at pH 7.5. Hiload Superdex 16/600 column and AKTA Pure (GE Healthcare) were used for further purification. Finally, dmCTPS was eluted with buffer containing 250 mM NaCl and 25 mM Tris HCl (pH 8.0).

### Cryo-EM Grid Preparation and Data Collection.

For preparing the substrate-bound dmCTPS sample, 1.2 μM dmCTPS protein was dissolved in the buffer with 25 mM Hepes, 2 mM UTP, 2 mM ATP, 2 mM GTP, 6 μM DON, and 10 mM MgCl_2_. For product-bound dmCTPS, 0.5 to 1 μM dmCTPS was dissolved in the buffer with 25 mM Hepes, 150 mM NaCl, 2 mM CTP, and 10 mM MgCl_2_. Samples were prepared with amorphous alloy film (No. M024-Au300-R12/13) and FEI Vitrobot (8 °C temperature, 3.5 s blotting time, −5 blot force). Images were taken with a Gatan K3 summit camera on an FEI Titan Krios electron microscope operated at 300 kV. The magnification was 22,500× in superresolution mode with the defocus rage −1.0 to −2.3 μm and a pixel size of 1.06 Å. The total dose was 60 e^−^/Å^2^ subdivided into 50 frames at 4-s exposure using SerialEM.

### Image Processing.

The whole workflow was done in Relion3.1-beta. Raw movies were dose weighted and aligned by MOTIONCOR2 through RELION3 GUI, and contrast transfer function (CTF) parameters were determined by CTFFIND4. Respectively, 424,195 and 1,563,553 particles were picked for dmCTPS^+Sub^ and dmCTPS^+Pro^ by autopicking. C1 and D2 symmetry was applied. After two-dimensional and three-dimensional (3D) classification, 107,556 and 344,869 particles were selected for the 3D refinement to generate two maps of 2.91 and 2.98 Å. CTF refinement and Bayesian polishing were applied to each particle. The 3D refinement and continued focus refinement with tight mask for the central tetramer were used in two different datasets. Finally, we constructed maps with resolution 2.48 Å for dmCTPS^+Sub^ and 2.65 Å for dmCTPS^+Pro^.

### Model Building and Refinement.

Our previous full-length *Drosophila* CTPS model [Protein Data Bank (PDB) ID: 6L6Z, 6LFG] was applied for the initial models of both datasets. Models of monomers were manually refined according to the electron density map with Coot software (38). The refined monomer models were symmetrized to build tetramer models in Chimera software (39). The tetramer models were subsequently real-space refined in Python-based hierarchical environment for integrated xtallography (Phenix) software (40).

### CTPS Activity Assays.

To measure the enzyme activity of dmCTPS in the presence of GTP at different levels, 2 μM dmCTPS was dissolved in the reaction buffer containing 25 mM Tris HCl (pH 7.5), 10 mM MgCl_2_, 2 mM ATP, 2 mM UTP, and 0, 0.2, and 2 mM of GTP. To initiate reaction, 10 mM glutamine was added into the mixture. The production of CTP was determined by measuring the absorption of a wavelength of 291 nm (15). Absorption of a wavelength of 291 nm of each reaction mixture was measured with SpectraMax i3 as the indication for CTP levels at individual time points.

### Tunnel Modeling.

The software CAVER Analyst59 was used to model tunnels through dmCTPS^+Sub^ and dmCTPS^+Pro^ models. A site adjacent to the Cys399 was used as starting coordinates for both models. Probe radii of 1.0 Å was used for both structures. The diameter and distance of the tunnel were also measured with CAVER Analyst.

## Supplementary Material

Supplementary File

Supplementary File

## Data Availability

Structural data have been deposited in PDB (PDB ID: 7DPT, 7DPW) and in Electron Microscopy Data Bank (EMDB ID: EMD-30810, EMD-30811).
