# Supplementary material for: Structural basis for ligand binding modes of CTP synthase
Source: Proc Natl Acad Sci U S A. 2021 Jul 23;118(30):e2026621118. doi: 10.1073/pnas.2026621118 (PMC8325340; doi:10.1073/pnas.2026621118)
Supplement: Supplementary File [file pnas.2026621118.sapp.pdf]

Structural basis for ligand binding modes of CTP synthase

Xian Zhou[1,]†, Chen-Jun Guo[1,2,3,]†, Chia-Chun Chang[1]†, Jiale Zhong[1,2,3], Huan-Huan Hu[1,2,3], Guang-Ming Lu[1,2,3], and Ji-Long Liu[1]*

SUPPLEMENTAL INFORMATION

SUPPLEMENTAL TABLE S1 AND FIGURES S1-S12

SUPPLEMENTAL MOVIE S1

Table S1. Cryo-EM data collection and model refinement

	dmCTPS+Sub (EMD-30810, PDB 7DPT)	dmCTPS+Pro (EMD-30811, PDB 7DPW)
**Data collection**		
EM equipment	Titan Krios	Titan Krios
Detector	K3 camera	K3 camera
Magnification	22,500x	22,500x
Voltage (kV)	300	300
Electron exposure ((e–/Å^2))	60	60
Defocus range(μm)	-1.0 to -2.3	-1.0 to -2.3
Pixel size(Å)	0.53	0.53
Symmetry imposed	D2	D2
Number of collected movies	2878	2259
Initial particle images (no.)	424195	1563553
Final particle images (no.)	107556	344869
Map resolution (Å)	2.5	2.7
FSC threshold	0.143	0.143
Map resolution range (Å)	2.4 - 3.8	2.5 - 4.7
**Refinement**		
Initial model used (PDB code)	6L6Z	6LFG
Map sharpening B-factor(Å^2)	-77	-90
Model composition		
Non-hydrogen atoms	18036	17812
Protein residues	2224	2224
Ligands	UTP,ATP,GTP,DON	CTP
Waters	76	32
Ions	12	8
B factors(Å^2)		
Protein	97	68
Ligand	88	32
Water	87	38
R.m.s. deviations		
Bond lengths (Å)	0.006	0.008
Bond angles (°)	0.663	0.81
Validation		
MolProbity score	1.69	2.56
Clashscore	3.24	7.84
Poor rotamers (%)	1.44	8.02
Ramachandran plot		
Favored (%)	92.78	93.14
Allowed (%)	7.04	6.68
Disallowed (%)	0.18	0.18

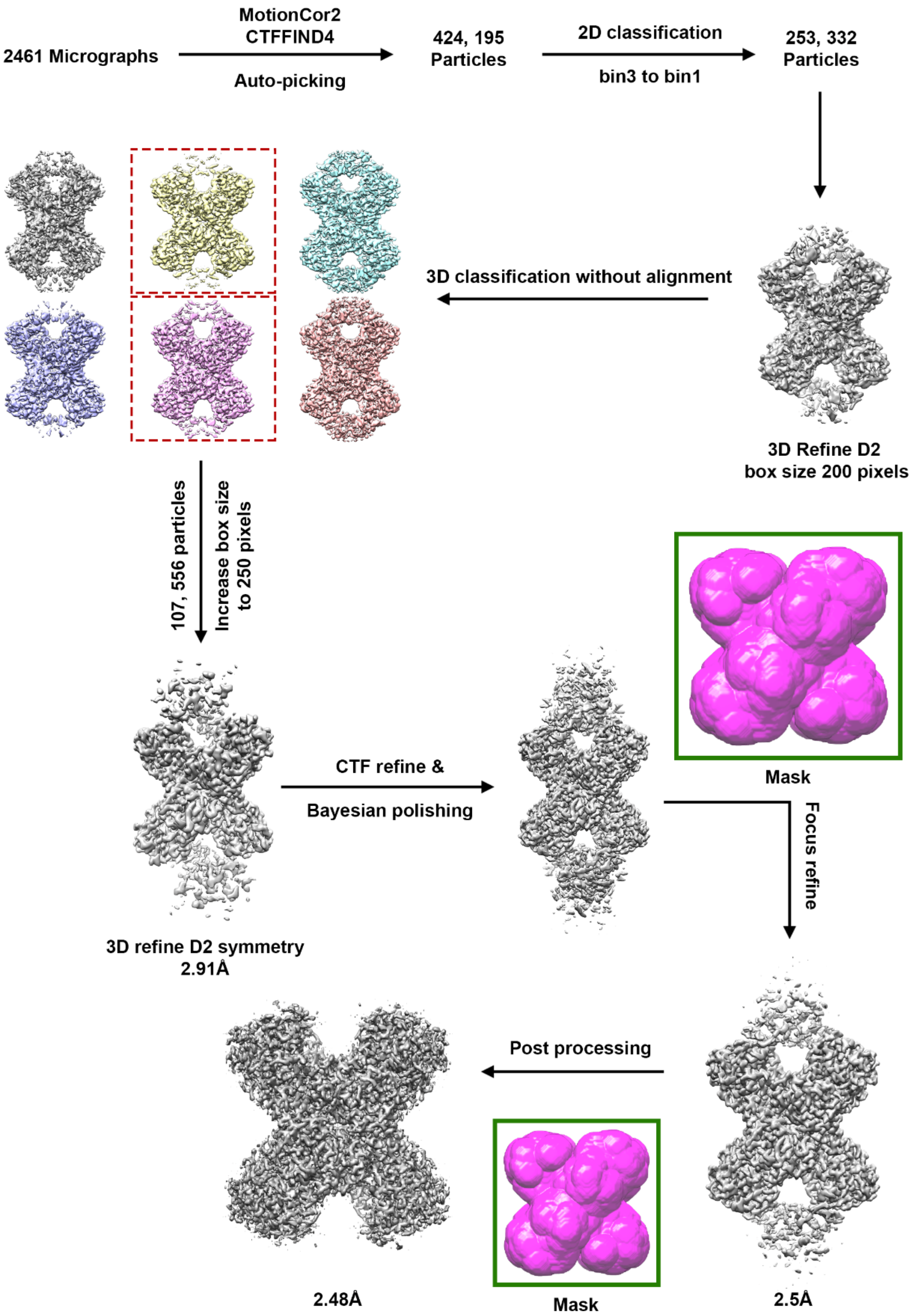

Figure S1. Image processing work flow of substrate-bound dmCTPS tetramer. In the reconstruction of the dmCTPS^{+Sub} tetramer, we collected 2461 micrographs and 253,332 particles were kept after 2D classification. After re-extraction of particles of dmCTPS^{+Sub} tetramer, different reconstructions were performed to generate the final map and model.

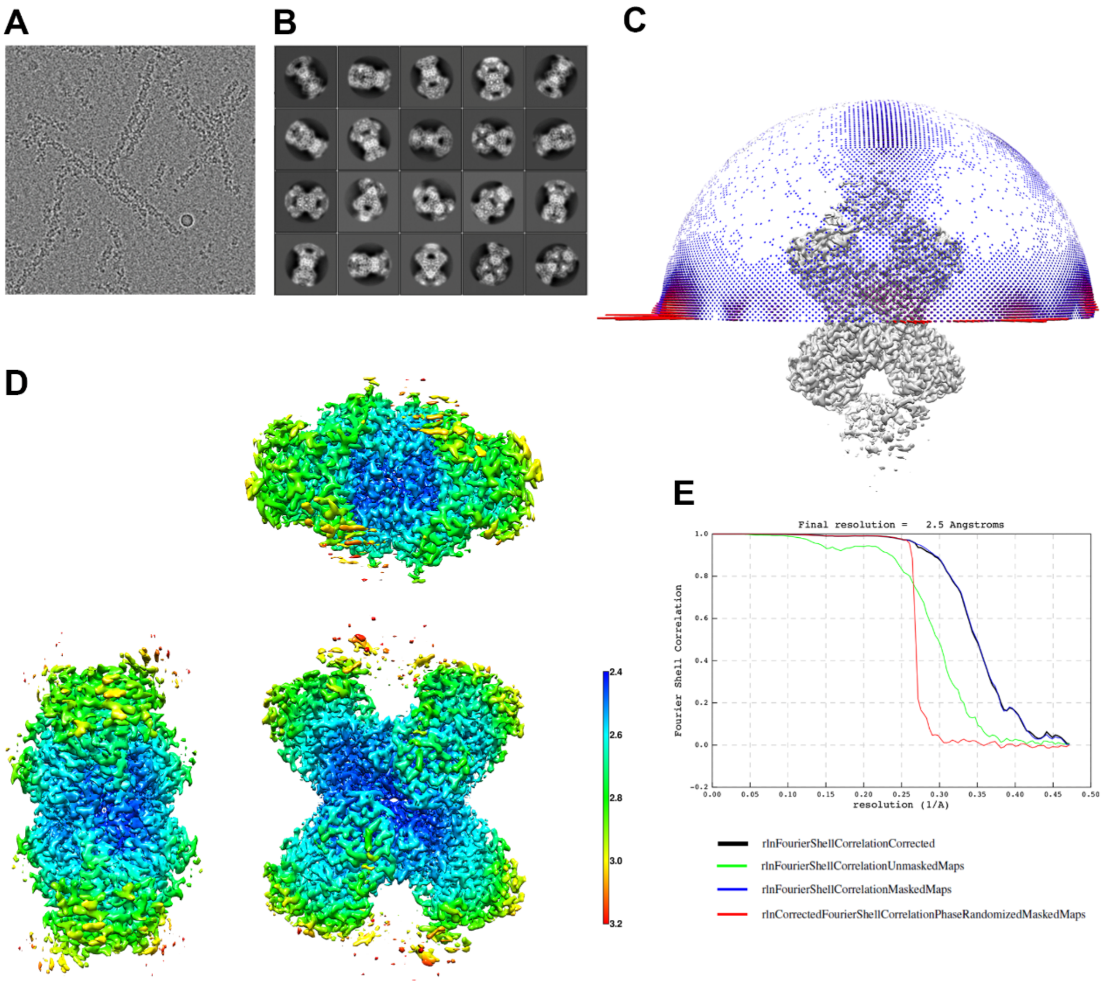

Figure S2. Statistics of the final density maps of the dmCTPS^{+Sub} tetramer. a, A representative cryo-EM micrograph of dmCTPS^{+Sub} tetramer/polymer. **b**, Representative 2D class averages of different views of the porcine dmCTPS^{+Sub} tetramer. **c**, Particle orientation distributions of the dmCTPS^{+Sub} tetramer in the last iteration of the 3D auto-refinement. **d**, Local resolution map of the dmCTPS^{+Sub} tetramer final 3D density map. **e**, Gold-standard FSC curve of the final density maps of the dmCTPS^{+Sub} tetramer.

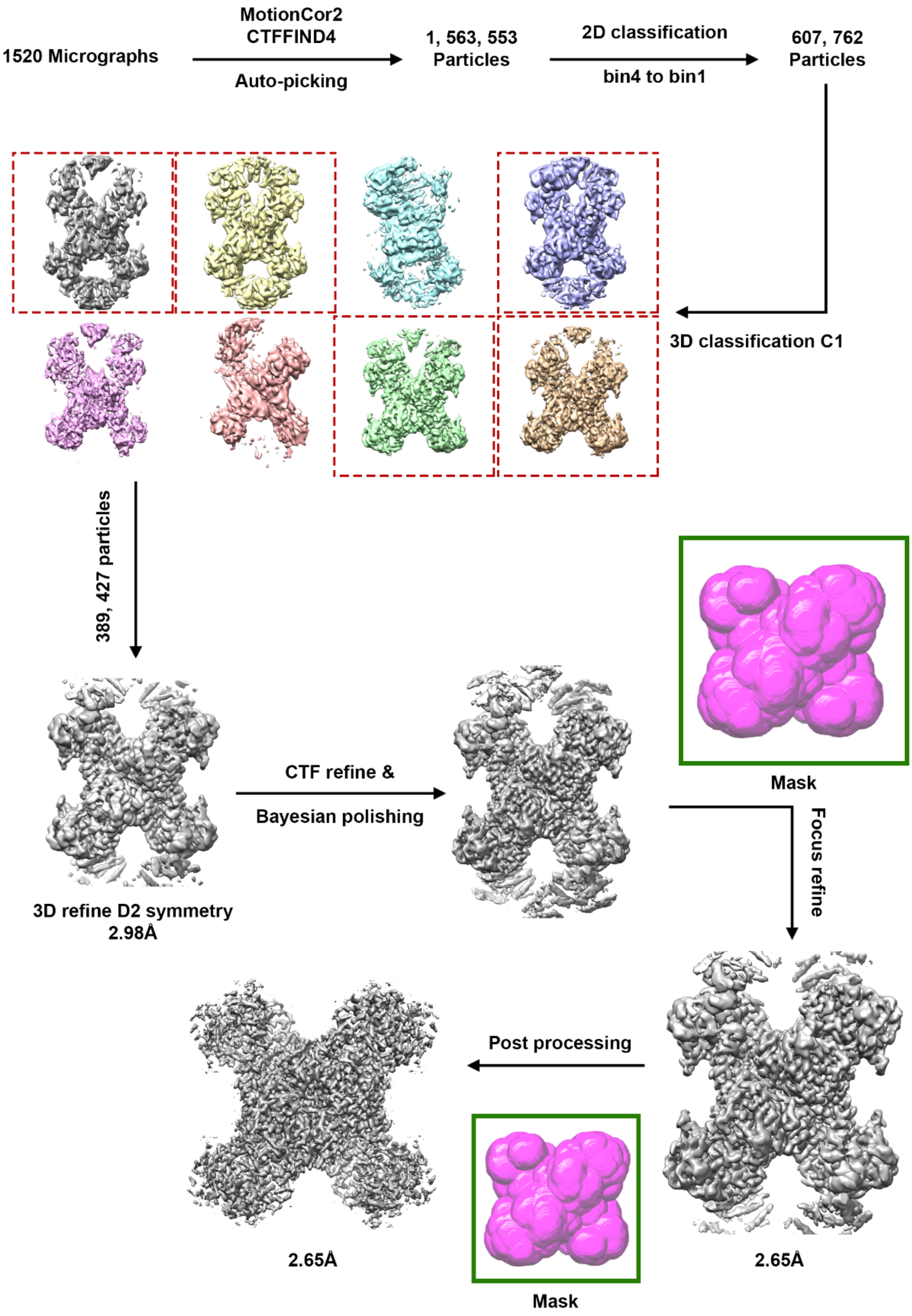

Figure S3. Image processing work flow of product-bound dmCTPS tetramer. In the reconstruction of the dmCTPS^{+Pro} tetramer, we collected 1520 micrographs and 607,762 particles were kept after 2D classification. After re-extraction of particles of dmCTPS^{+Pro} tetramer, different reconstructions were performed to generate the final map and model.

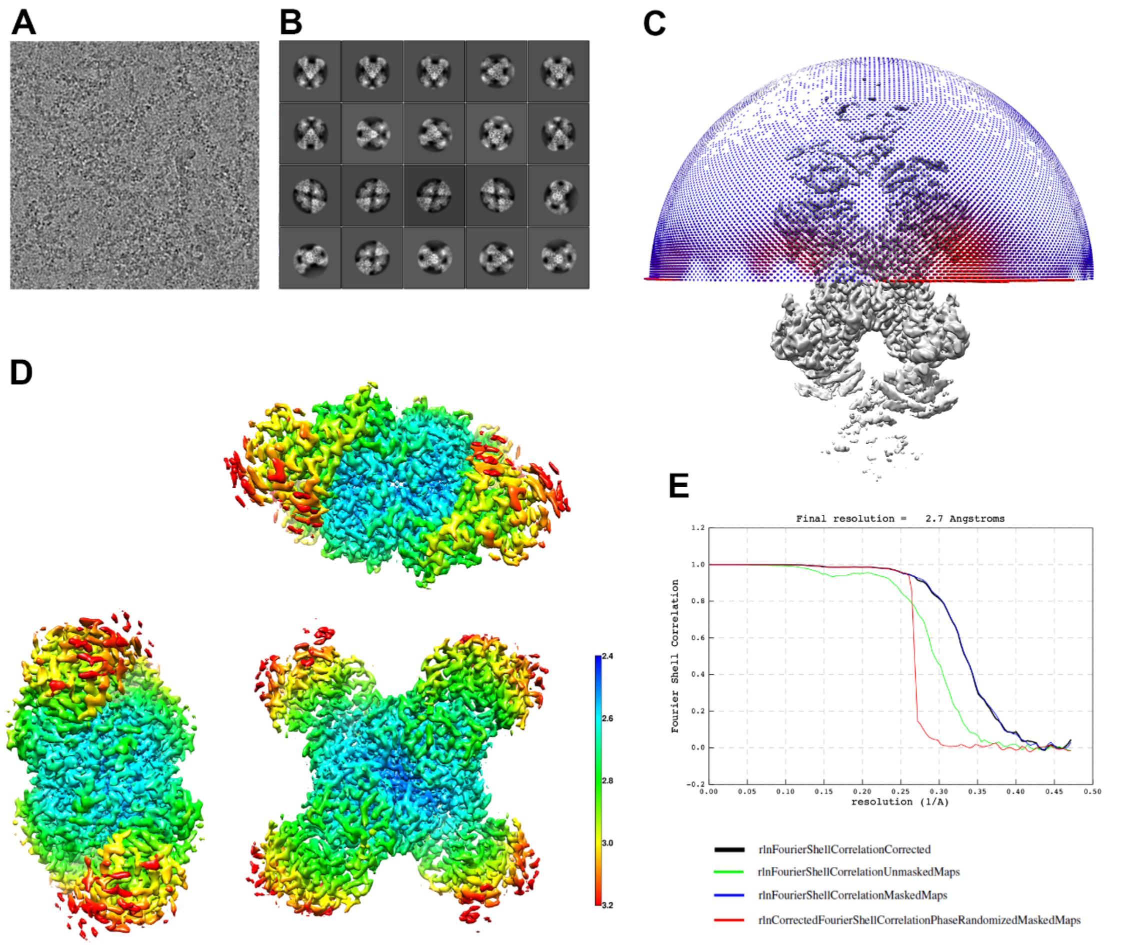

Figure S4. Statistics of the final density maps of the dmCTPS^{+Pro} tetramer. a, A representative cryo-EM micrograph of dmCTPS^{+Pro} tetramer/polymer. **b**, Representative 2D class averages of different views of the porcine dmCTPS^{+Pro} tetramer. **c**, Particle orientation distributions of the dmCTPS^{+Pro} tetramer in the last iteration of the 3D auto-refinement. **d**, Local resolution map of the dmCTPS^{+Pro} tetramer final 3D density map. **e**, Gold-standard FSC curve of the final density maps of the dmCTPS^{+Pro} tetramer.

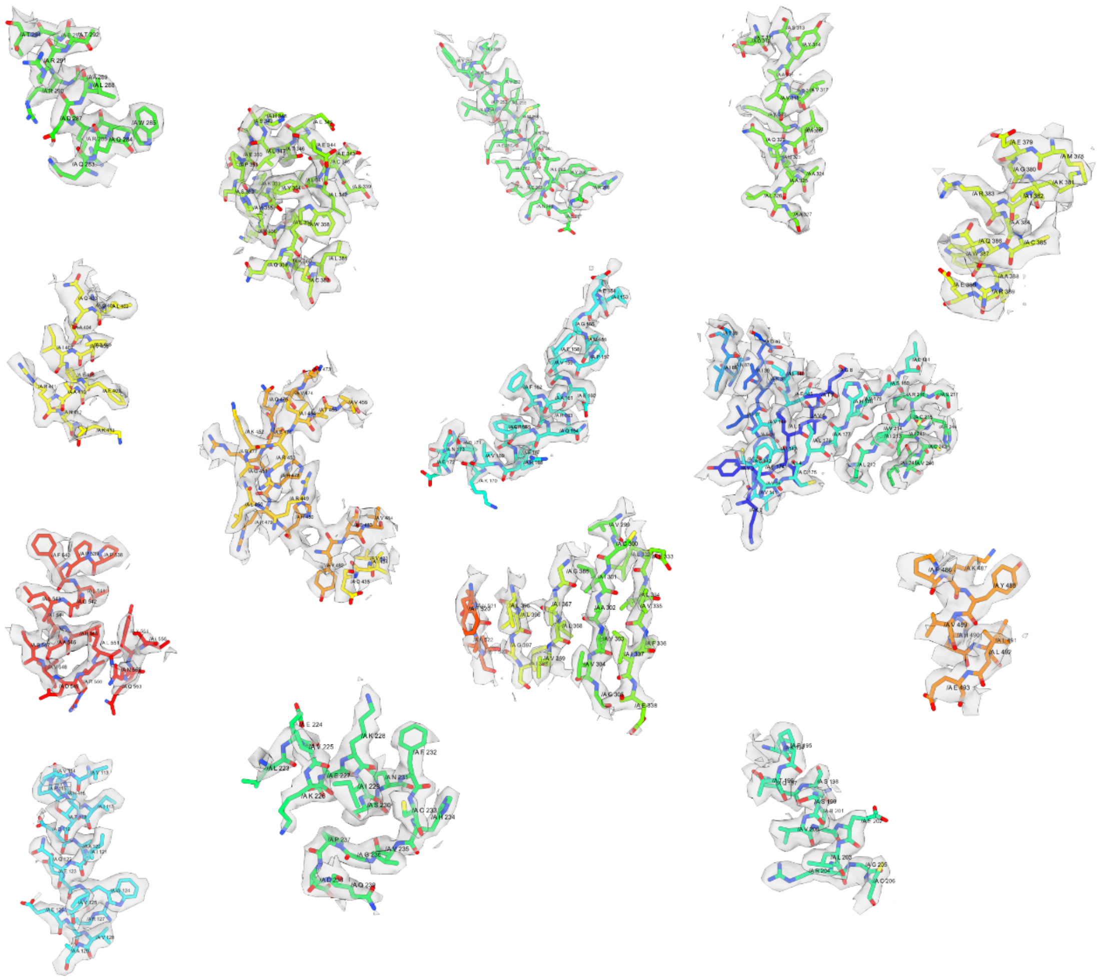

Figure S5. Representative electron density map of individual regions of dmCTPS^{+Sub} model.

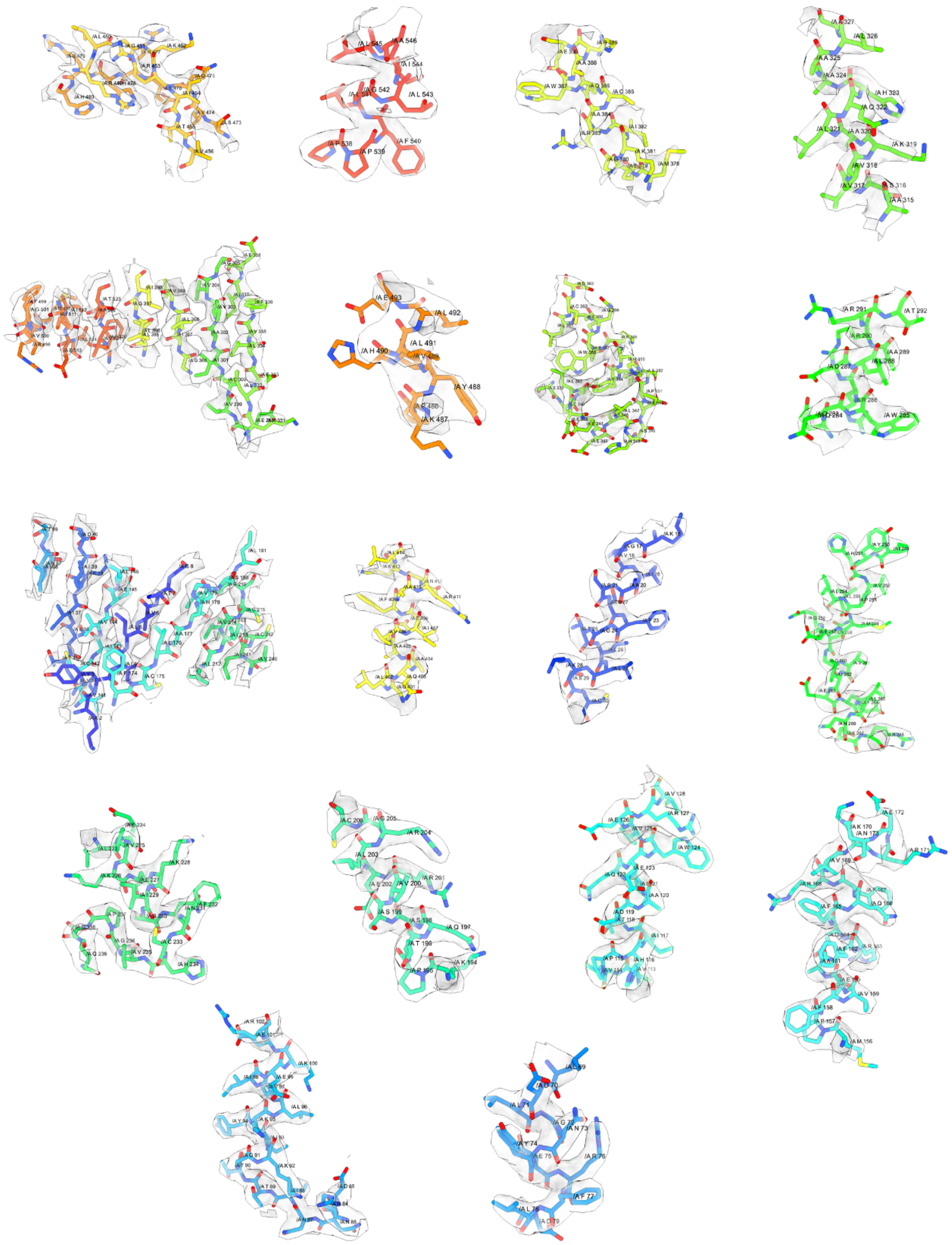

Figure S6. Representative electron density map of individual regions of dmCTPS^{+Pro} model.

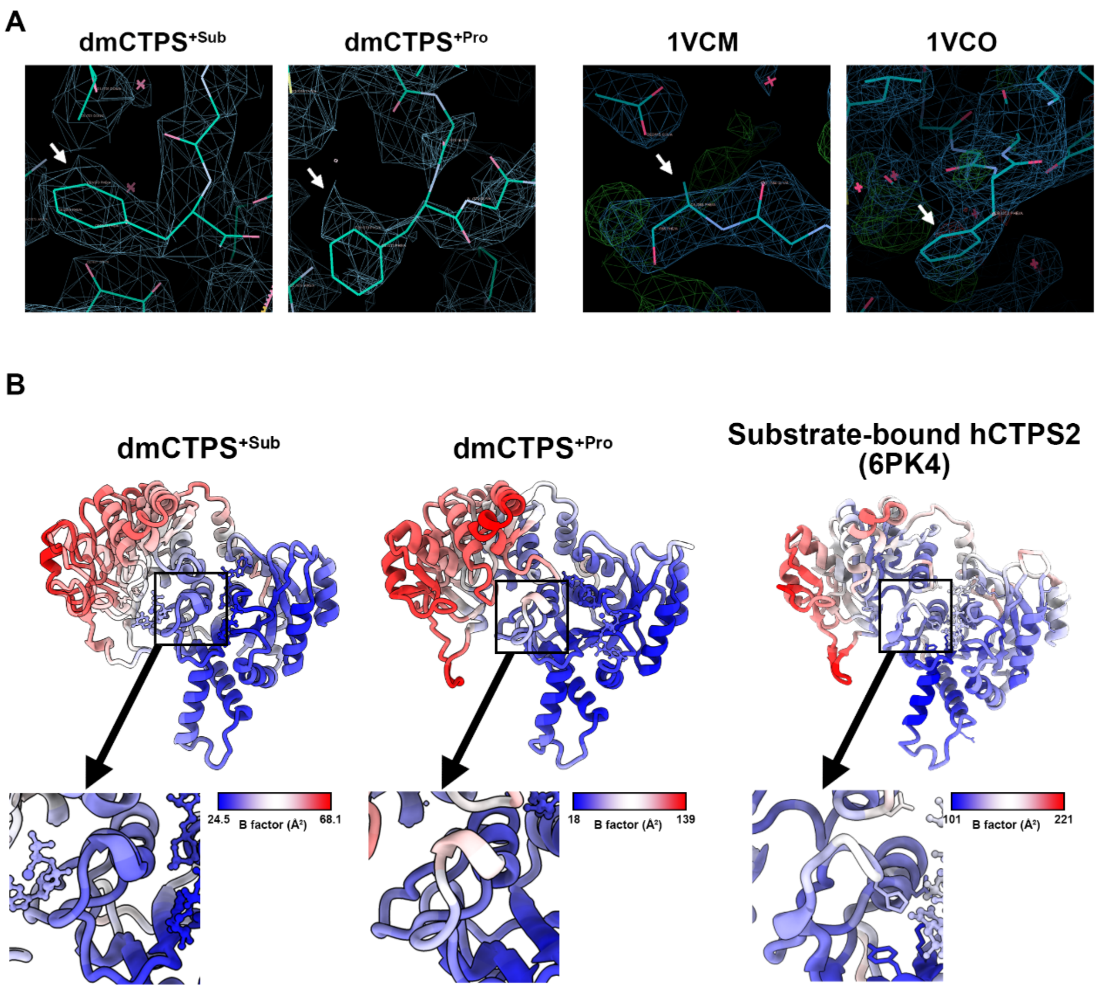

Figure S7. The comparisons of electron density maps of dmCTPS with reported CTPS models. a, The electron density maps of Phe373 in dmCTPS^{+Sub}, dmCTPS^{+Pro}, and of Phe365 in *Thermus thermophilus* CTPS in the apo state (1VCM) and the glutamine-bound state (1VCO). Phe373 in dmCTPS and Phe365 in ttCTPS are indicated by arrows. **b**, B factors are shown on monomers of dmCTPS^{+Sub}, dmCTPS^{+Pro} and substrate-bound hCTPS2 (6PK4) models. The region of the ammonia tunnel is highlighted.

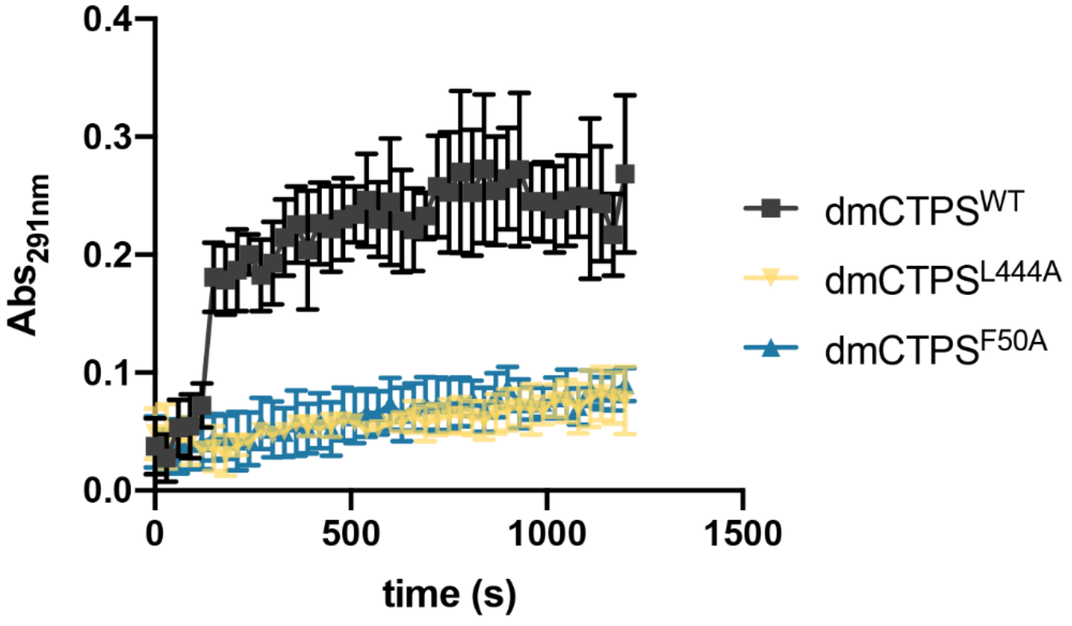

Figure S8. Analysis for generation of CTP by mutant dmCTPS (F50A and L444A) in condition with 2 mM of GTP.

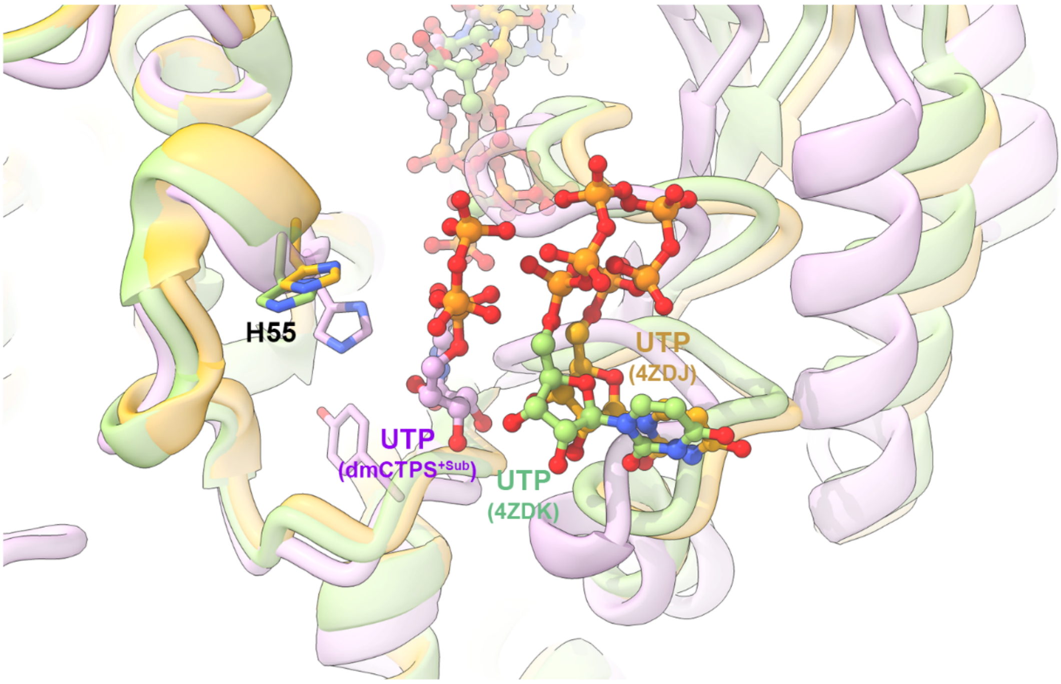

Figure S9. The structure comparison indicates the difference of distance between His55 and UTP in the open and closed CTPS conformations. The dmCTPS^{+Sub} model is shown in pink, the models of *Mycobacterium tuberculosis* CTPS (4ZDK, bound with UTP, ACP and DON) and (4ZDJ, bound with UTP) are shown in green and yellow, respectively.

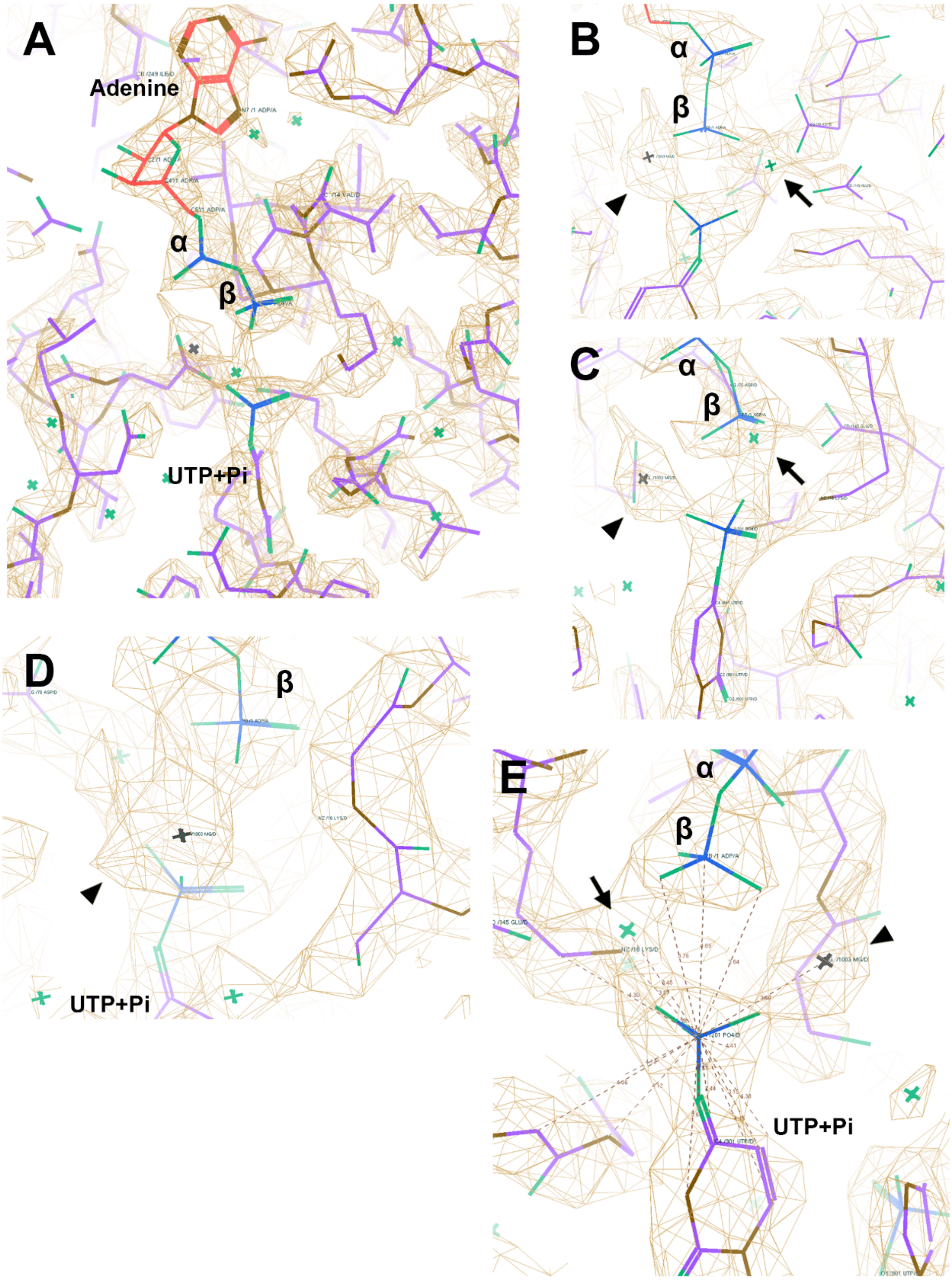

Figure S10. The electron density map of ligands at the AL domain active site of substrate-bound state. a, The general map of ligands at the AL domain when the map level was set as 10σ. In the map the α-phosphate and β-phosphate of the ATP match the density very well. However, the γ-phosphate of the ATP is not seen with the same setting. Meanwhile, a large density appears to be connected with the uracil of the UTP. **b**, **c**, There are two other density groups are located at both sides of the β-phosphate of ADP (indicated by arrow and arrowhead). Although the density indicated by the arrow

is connected with the β-phosphate, its size is too small to be a phosphate. In addition, it is also connected with Asp70, Glu145 and the additional density on the uracil, making it unlikely a phosphate. **d,** The density indicated by the arrowhead displays a density extension under lower map level (7.2σ), implying the presence of six bound water molecules. According to the catalytic process, we suggest the additional density on the uracil is the γ-phosphate of the ATP transferred to the 4-oxygen atom of the uracil base and the arrow and arrowhead indicate a water and a Mg^{2+} ion, respectively. **e,** The distance from the β-phosphate of ADP to the phosphate on the uracil is about 4.09 Å, far larger than the ordinary distance between β- and γ-phosphate. Meanwhile, the Mg^{2+} is slightly merged with the phosphate on the uracil. These suggest that transfer of γ-phosphate which is catalyzed by the Mg^{2+} is captured in our model.

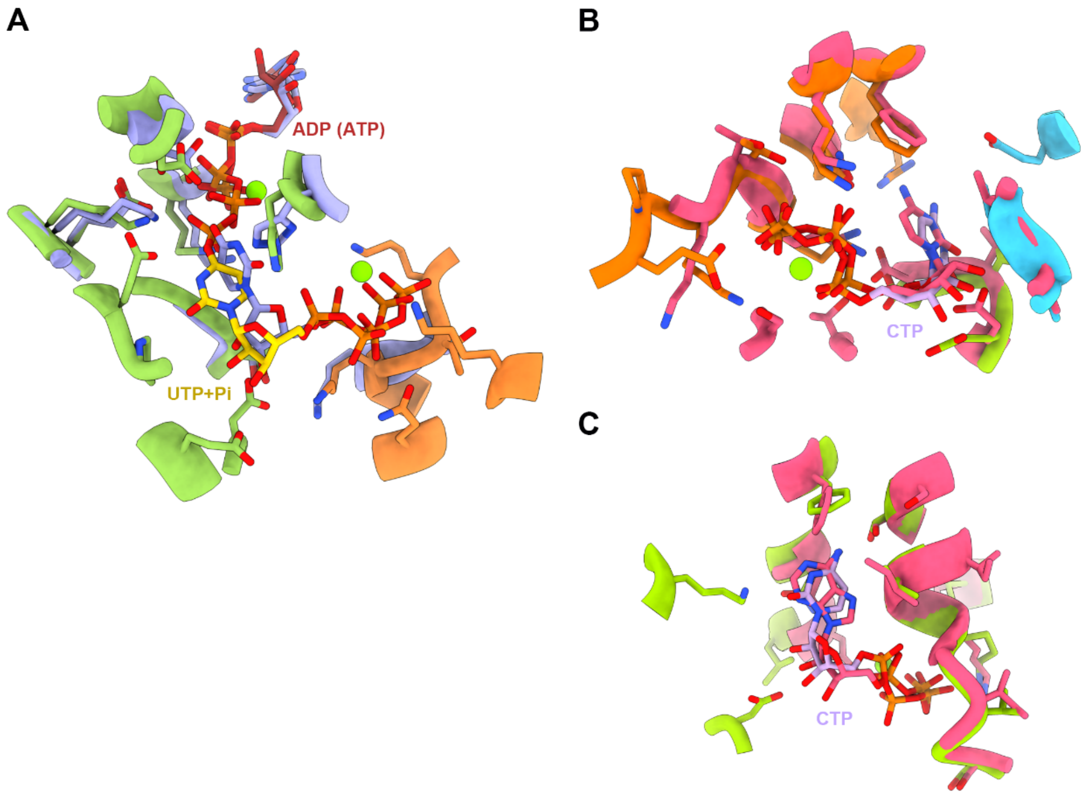

Figure S11. The comparison of dmCTPS and hCTPS2 at ATP/ADP, UTP and CTP binding sites. **a**, The structural comparison of dmCTPS^{+Sub} and substrate-bound hCTPS2 (6PK4) shows similar binding modes of ATP/ADP and UTP. **b** and **c**, The structural comparison of dmCTPS^{+Pro} and product-bound hCTPS2 (6PK7) shows the binding modes of CTP at UTP binding site (**b**) and ADP binding site (**c**).

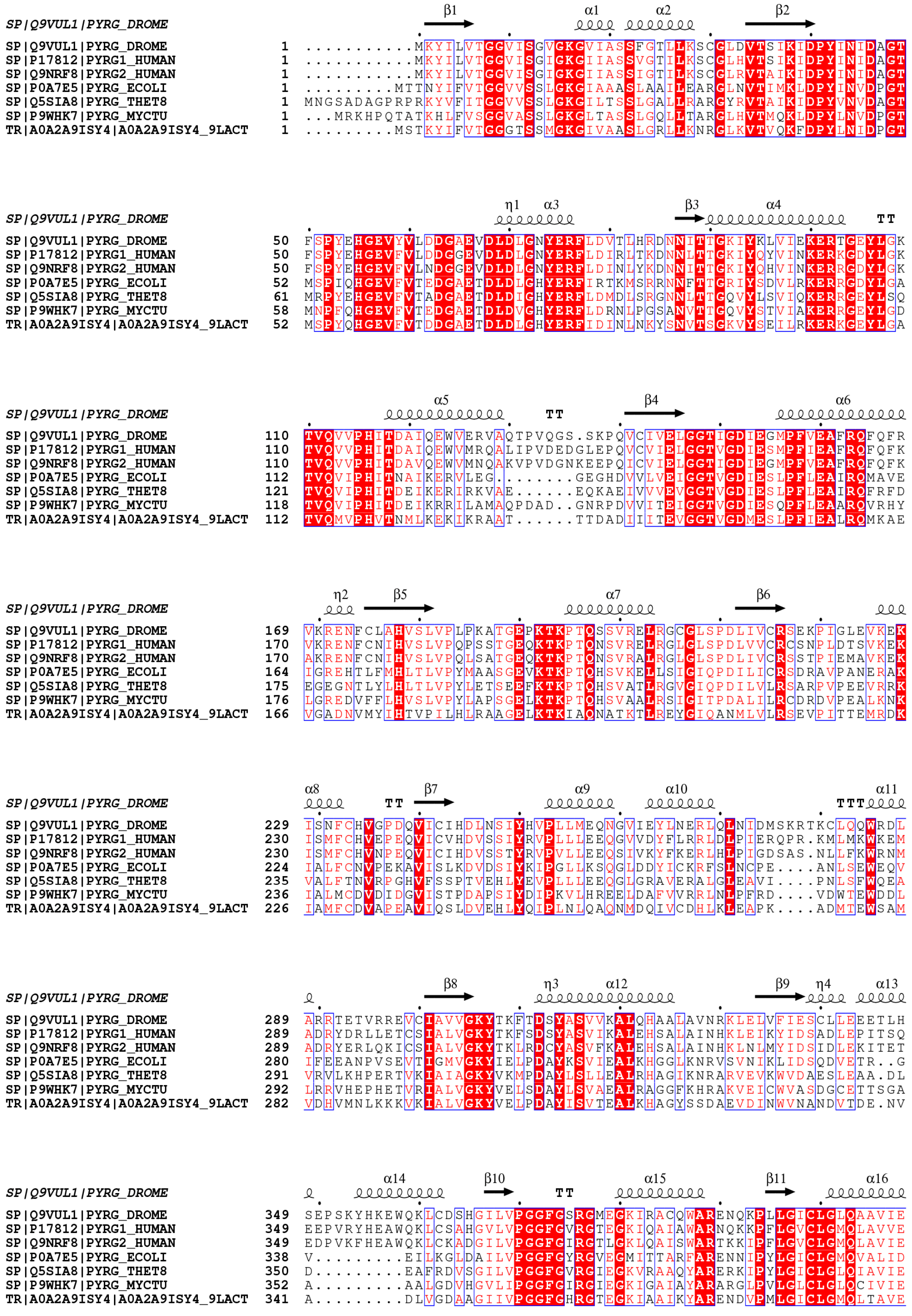
SP|Q9VUL1|PYRG_DROME β1 α1 α2 β2
SP|Q9VUL1|PYRG_DROME 1 ...........MKYILVTGGVISGVGKGVIASSFGTLLKSCGLDVTSIKIDPYINIDAGT
SP|P17812|PYRG1_HUMAN 1 ...........MKYILVTGGVISGIGKGIIASSVGTILKSCGLHVTSIKIDPYINIDAGT
SP|Q9NRF8|PYRG2_HUMAN 1 ...........MKYILVTGGVISGIGKGIIASSIGTILKSCGLRVTAIKIDPYINIDAGT
SP|P0A7E5|PYRG_ECOLI 1 .........MTTNYIFVTGGVVSSLGKGIAAASLAAILEARGLNVTIMKLDPYINVDPGT
SP|Q5SIA8|PYRG_THET8 1 MNGSADAGPRPRKYVFITGGVVSSLGKGILTSSLGALLRARGYRVTAIKIDPYVNVDAGT
SP|P9WHK7|PYRG_MYCTU 1 ...MRKHPQTATKHLFVSGGVASSLGKGLTASSLGQLLTARGLHVTMQKLDPYLNVDPGT
TR|A0A2A9ISY4|A0A2A9ISY4_9LACT 1 .........MSTKYIFVTGGTSSMGKGIVAASLGRLLKNRGLKVTVQKFDPYLNIDPGT
SP|Q9VUL1|PYRG_DROME η1 α3 β3 α4 TT
SP|Q9VUL1|PYRG_DROME 50 FSPYEHGEVYVLDDGAEVDLDLGNYERFLDVTLHRDNNITTGKIYKLVIEKERTGEYLGK
SP|P17812|PYRG1_HUMAN 50 FSPYEHGEVFVLDDGGEVDLDLGNYERFLDIRLTKDNNLTTGKIYQYVINKERKGDYLGK
SP|Q9NRF8|PYRG2_HUMAN 50 FSPYEHGEVFVLNDGGEVDLDLGNYERFLDINLYKDNNITTGKIYQHVINKERRGDYLGK
SP|P0A7E5|PYRG_ECOLI 52 MSPIQHGEVFVTEDGAETDLDLGHYERFIRTKMSRRNNFTTGRIYSDVLRKERRGDYLGA
SP|Q5SIA8|PYRG_THET8 61 MRPYEHGEVFVTADGAETDLDIGHYERFLDMDLSRGNNLTTGQVYLSVIQKERRGEYLSQ
SP|P9WHK7|PYRG_MYCTU 58 MNPFQHGEVFVTEDGAETDLDVGHYERFLDRNLPGSANVTTGQVYSTVIAKERRGEYLGD
TR|A0A2A9ISY4|A0A2A9ISY4_9LACT 52 MSPYQHGEVFVTDDGAETDLDLGHYERFIDINLNKYSNVTSGKVYSEILRKERKGEYLGA
SP|Q9VUL1|PYRG_DROME α5 TT β4 α6
SP|Q9VUL1|PYRG_DROME 110 TVQVVPHITDAIQEWVERVAQTPVQGS.SKPQVCIVELGGTIGDIEGMPFVEAFRQFQFR
SP|P17812|PYRG1_HUMAN 110 TVQVVPHITDAIQEWVMRQALIPVDEDGLEPQVCVIELGGTVGDIESMPFIEAFRQFQFK
SP|Q9NRF8|PYRG2_HUMAN 110 TVQVVPHITDAVQEWVMNQAKVPVDGNKEEPQICVIELGGTIGDIEGMPFVEAFRQFQFK
SP|P0A7E5|PYRG_ECOLI 112 TVQVIPHITNAIKERVLEG........GEGHDVVLVEIGGTVGDIESLPFLEAIRQMAVE
SP|Q5SIA8|PYRG_THET8 121 TVQVIPHITDEIKERIRKVAE......EQKAEIVVVEVGGTVGDIESLPFLEAIRQFRFD
SP|P9WHK7|PYRG_MYCTU 118 TVQVIPHITDEIKRRILAMAQPDAD..GNRPDVVITEIGGTVGDIESQPFLEAARQVRHY
TR|A0A2A9ISY4|A0A2A9ISY4_9LACT 112 TVQMVPHVTNMLKEKIKRAAT......TTDADIIITEVGGTVGDMESLPFIEALRQMKAE
SP|Q9VUL1|PYRG_DROME η2 β5 α7 β6
SP|Q9VUL1|PYRG_DROME 169 VKRENFCLAHVSLVPLPKATGEPKTKPTQSSVRELRGCGLSPDLIVCRSEKPIGLEVKEK
SP|P17812|PYRG1_HUMAN 170 VKRENFCNIHVSLVPQPSSTGEQKTKPTQNSVRELRGLGLSPDLVVCRCSNPLDTSVKEK
SP|Q9NRF8|PYRG2_HUMAN 170 AKRENFCNIHVSLVPQLSATGEQKTKPTQNSVRALRGLGLSPDLIVCRSSTPIEMAVKEK
SP|P0A7E5|PYRG_ECOLI 164 IGREHTLFMHLTLVPYMAASGEVKTKPTQHSVKELLSIGIQPDILICRSDRAVPANERAK
SP|Q5SIA8|PYRG_THET8 175 EGEGNTLYLHLTLVPYLETSEEFKTKPTQHSVATLRGVGIQPDILVLRSARPVPEEVRRK
SP|P9WHK7|PYRG_MYCTU 176 LGREDVFFLHVSLVPYLAPSGELKTKPTQHSVAALRSIGITPDALILRCDRDVPEALKNK
TR|A0A2A9ISY4|A0A2A9ISY4_9LACT 166 VGADNVMYIHTVPILHLRAAGELKTKIAQNATKTLREYGIQANMLVLRSEVPITTEMRDK
SP|Q9VUL1|PYRG_DROME α8 TT β7 α9 α10 TTT α11
SP|Q9VUL1|PYRG_DROME 229 ISNFCHVGPDQVICIHDLNSIYHVPLLMEQNGVIEYLNERLQLNIDMSKRTKCLQQWRDL
SP|P17812|PYRG1_HUMAN 230 ISMFCHVEPEQVICVHDVSSIYRVPLLLEEQGVVDYFLRRLDLPIERQPR.KMLMKWKEM
SP|Q9NRF8|PYRG2_HUMAN 230 ISMFCHVNPEQVICIHDVSSTYRVPVLLEEQSIVKYFKERLHLPIGDSAS.NLLFKWRNM
SP|P0A7E5|PYRG_ECOLI 224 IALFCNVPEKAVISLKDVDSIYKIPGLLKSQGLDDYICKRFSLNCPE....ANLSEWEQV
SP|Q5SIA8|PYRG_THET8 235 VALFTNVRPGHVFSSPTVEHLYEVPLLLEEQGLGRAVERALGLEAVI....PNLSFWQEA
SP|P9WHK7|PYRG_MYCTU 236 IALMCDVDIDGVISTPDAPSIYDIPKVLHREELDAFVVRRLNLPFRD....VDWTEWDDL
TR|A0A2A9ISY4|A0A2A9ISY4_9LACT 226 IAMFCDVAPEAVIQSLDVEHLYQIPLNLQAQNMDQIVCDHLKLEAPK....ADMTEWSAM
SP|Q9VUL1|PYRG_DROME β8 η3 α12 β9 η4 α13
SP|Q9VUL1|PYRG_DROME 289 ARRTETVRREVCIAVVGKYTKFTDSYASVVKALQHAALAVNRKLELVFIESCLLEEETLH
SP|P17812|PYRG1_HUMAN 289 ADRYDRLLETCSIALVGKYTKFSDSYASVIKALEHSALAINHKLEIKYIDSADLEPITSQ
SP|Q9NRF8|PYRG2_HUMAN 289 ADRYERLQKICSIALVGKYTKFSDCYASVFKALEHSALAINHKLNLMYIDSIDLEKITET
SP|P0A7E5|PYRG_ECOLI 280 IFEEANPVSEVTIGMVGKYIELPDAYKSVIEALKHGGLKNRVSVNIKLIDSQDVETR..G
SP|Q5SIA8|PYRG_THET8 291 VRVLKHPERTVKIAIAGKYVKMPDAYLSLLEALRHAGIKNRARVEVKWVDAESLEAA.DL
SP|P9WHK7|PYRG_MYCTU 292 LRRVHEPHETVRIALVGKYVELSDAYLSVAEALRAGGFKHRAKVEICWVASDGCETTSGA
TR|A0A2A9ISY4|A0A2A9ISY4_9LACT 282 VDHVMNLKKKVKIALVGKYVELPDAYISVTEALKHAGYSSDAEVDINWVNANDVTDE.NV
SP|Q9VUL1|PYRG_DROME α14 β10 TT α15 β11 α16
SP|Q9VUL1|PYRG_DROME 349 SEPSKYHKEWQKLCDSHGILVPGGFGSRGMEGKIRACQWARENQKPLLGICLGLQAAVIE
SP|P17812|PYRG1_HUMAN 349 EEPVRYHEAWQKLCSAHGVLVPGGFGVRGTEGKIQAIAWARNQKKPFLGVCLGMQLAVVE
SP|Q9NRF8|PYRG2_HUMAN 349 EDPVKFHEAWQKLCKADGILVPGGFGIRGTLGKLQAISWARTKKIPFLGVCLGMQLAVIE
SP|P0A7E5|PYRG_ECOLI 338 V.........EILKGLDAILVPGGFGYRGVEGMITTARFARENNIPYLGICLGMQVALID
SP|Q5SIA8|PYRG_THET8 350 D.........EAFRDVSGILVPGGFGVRGIEGKVRAAQYARERKIPYLGICLGLQIAVIE
SP|P9WHK7|PYRG_MYCTU 352 A.........AALGDVHGVLIPGGFGIRGIEGKIGAIAYARARGLPVLGLCLGLQCIVIE
TR|A0A2A9ISY4|A0A2A9ISY4_9LACT 341 A.........DLVGDAAGIIVPGGFGHRGTEGKIAAIKYARENDVPMLGICLGMQLTAVE

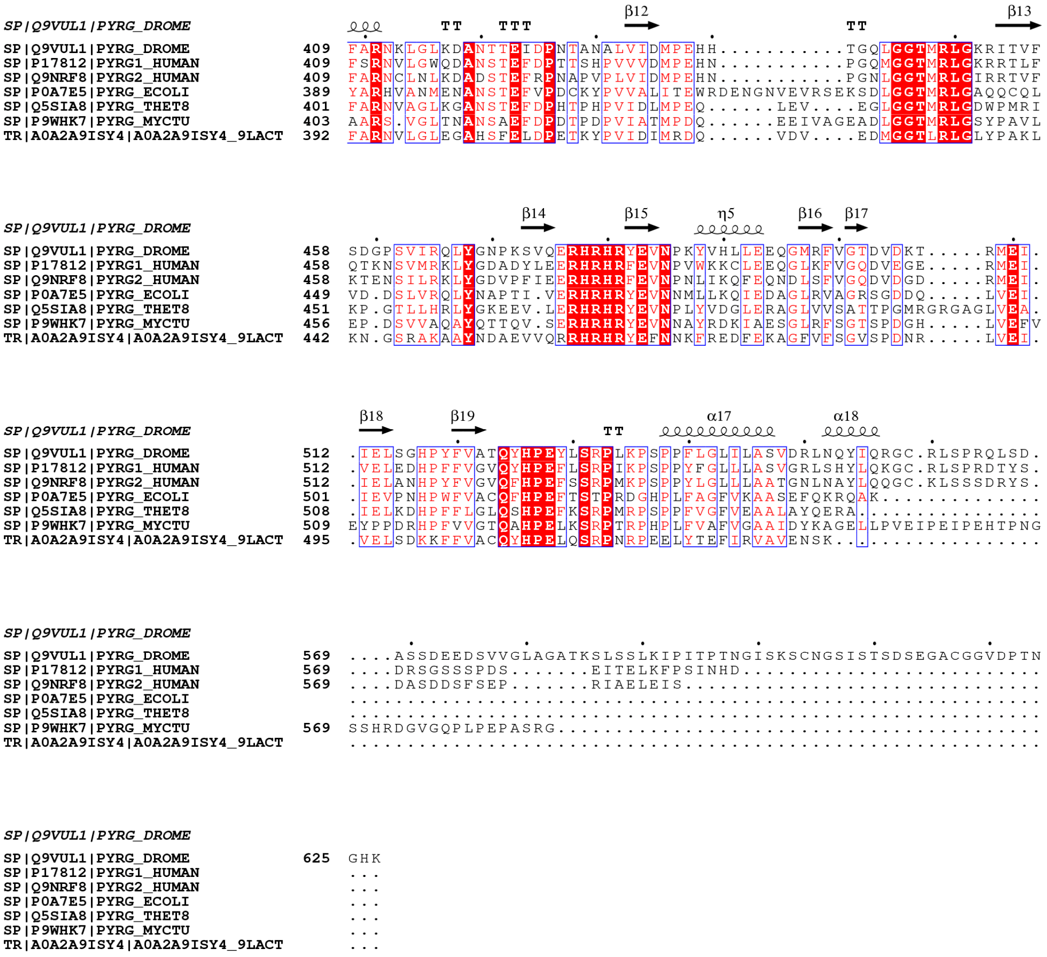

Figure S12. Comparison of CTPS sequences of various organisms.

Legends for SI Appendix videos

SUPPLEMENTAL MOVIE S1. Animation of CTPS morphing from the CTP-state to DON-state tetramer conformations. The movie displays the dmCTPS structure morphed from product-bound to substrate-bound state. Colored by monomer.
